# Report on the Progress of Practical Medicine, in the Departments of Midwifery and the Diseases of Women and Children, in the Years 1845-6

**Published:** 1847-01

**Authors:** Charles West

**Affiliations:** Member of the Royal College of Physicians, Physician-Accoucheur and Lecturer on Midwifery to the Middlesex Hospital, and Senior Physician to the Royal Infirmary for Children


### PART FOURTH.

### Original Reports and Memoirs.

### REPORT ON THE PROGRESS OF PRACTICAL MEDICINE,

IN THE DEPARTMENTS OF

### MIDWIFERY AND THE DISEASES OF WOMEN AND CHILDREN,

IN THE YEARS 1845-6.

By Charles West, M.D.,

Member of the Royal College of Physicians, Physician-Accoucheur and Lecturer on Midwifery to the Middlesex Hospital, and Senior Physician to the Royal Infirmary for Children.

This Report refers to a period of fifteen months extending from the 1st of July, 1845, to the 30th of September, 1846; and its general arrangement differs in no respect from that followed in the two previous Reports.

### 1. On the Progress of Midwifery.

A reprint of Dr. Murphy's Lectures on Parturition, which were originally published in the 'Lancet,' is the only new work that has appeared on this subject. Dr. Rigby's Lectures on Midwifery are now in course of publication in the 'Medical Times,' and Dr. J. H. Davis has contributed to the 'Lancet' very numerous papers on the management of difficult and preternatural labour.

#### PREGNANCY.

*Signs of Pregnancy.* Dr. Mikschick* has been led, by the examination of the urine of 50 pregnant women, to the same conclusion as many other investigators have already arrived at, with reference to the little value to be attached to the presence of kysteine in the urine as a sign of pregnancy. He found that in the majority of cases, an opalescent membrane formed on the surface of the urine after it had been allowed to stand for several days, but the same appearance was observed in many other instances independent of either lactation or pregnancy.

*Disorders of Pregnancy.* Mr. H. B. Lane† relates a case of that rare occurrence, *anteversion of the pregnant uterus.* The patient was a woman 35 years old, who fell down stairs in the 6th week of her pregnancy, striking her back and hips. Soon afterwards she began to suffer from tenesmus, dysuria, and frequent desire to pass water, and a varicose condition of the veins at the entrance of the vagina came on. When her pregnancy was 3½ months advanced, her state became much aggravated, and she could no longer pass water except in the horizontal posture. The os uteri could not be reached,

* Oesterr. Med. Jahrb., Dec. 1845.

† Lancet, Sept. 27, 1845.

but a tumour was felt through the superior vaginal wall, encroaching on its cavity, and so firmly fixed as to be quite immoveable. The use of the catheter afforded much relief, and though an attempt at the reposition of the uterus failed, yet the organ two days afterwards returned to its proper position, and the patient went to the full term of her pregnancy without suffering any further inconvenience.

Dr. Skae* has recorded an instance of *inversion of the uterus*, occurring 10 days *after abortion* at the 4th month of pregnancy. Considerable hemorrhage had occurred on the third day after the miscarriage, in consequence of the patient attempting to move about; but the uterus became inverted during an attack of vomiting, which was attended with a sensation of something falling down within her, and was followed by prostration of strength, bearing down, and flooding.

She was seen 12 hours after the accident, when the os tincæ was open to the width of two inches, and dilatable, and a tumour passed through it into the vagina. After two efforts, each continued for 15 or 20 minutes, the tumour was returned within the os uteri; but the fundus of the organ was not thoroughly reverted till the following day. The patient had since menstruated naturally, and continued well. [A somewhat similar case is related by Lisfranc, 'Clin. Chirurg.' iii. 380; but the inversion of the uterus, which had probably existed for five years, was not discovered until after the patient's death.]

*Extra-uterine Pregnancy.* Dr. Cogswell† has related a case of supposed *ovarian pregnancy*, in which the symptoms subsided between the 2d and 3d month, but the woman subsequently conceived and gave birth to two living children. In her second labour, it became necessary to puncture a soft tumour, situated between the vagina and rectum, which refilled, and, having been again tapped, continued to discharge a dark fluid, until the patient died exhausted, three months after delivery. The sac was found to be formed by the enlarged left ovary, which contained a pint of fluid besides some fœtal hair and bones. The case described as ovarian pregnancy by Dr. Harris‡, though the account given of it is very imperfect, may yet be decided not to have been an instance of extra-uterine pregnancy, but of ovarian disease, the right ovary being dropsical; hair and a portion of bone having been formed in the interior of the left.

A case of *Fallopian pregnancy* is related by Mr. Allport,§ which terminated fatally by hemorrhage, consequent on rupture of the tube in the 5th month of pregnancy; and another is related by Dr. Oldham‖ in which the patient died from the same cause at the 3d month. The uterus was in both cases lined with decidua.

Dr. Oldham's second case appears to have been one of *interstitial pregnancy*, the ovum having been developed in the uterine substance just behind the end of the tube. The tube was impervious, both above and below the supposed situation of the ovum, which appeared to have occupied a cell in the uterine substance large enough to contain a horse chesnut. Some doubt, however, is thrown on the real nature of the case, by the circumstance that the ovum, which had probably escaped through the ruptured walls of the cell, could not be found, and that the corpus luteum, though distinctly marked, was found in the ovary opposite to that side of the uterus where the supposed ovum was situated.

References are given below to several cases of *abdominal pregnancy*.¶ In

* Northern Journal of Medicine, July, 1845.

† Boston Medical Journal, July, 1845.

‡ Southern (American) Journal, July, 1846.

§ Lancet, Oct. 18, 1845.

‖ Guy's Hospital Reports, 1845.

¶ Grossi, Annales de la Chirurgie, Sept., 1845; Jobert et Dubois, Gaz. des Hôpitaux, July 5, 1845; Stevens, Amer. Journal of Med. Science, July, 1845; Yardley, ibid., April, 1846; Whinery, ibid., April, 1846; Craddock, American Medical Examiner, May, 1846; Mason, ibid., Jan., 1846; Carganico, Med. Zeitung, Aug. 13, 1845; Cerise, Gaz. des Hôpit., Jan. 12, 1846; McCulloch, British American Journal, Oct., 1845; Götz, Oesterr. Med. Jahrbüch, April, 1846.

the case reported by Dr. Grossi, the patient was still living, and the symptoms were by no means conclusive, while it is to be regretted that no attempts were made to determine, by means of the stethoscope, whether certain movements perceived through the abdominal walls were really due to the presence of a fœtus. A somewhat similar doubt attends the case which was under the care of MM. Jobert and Dubois, though the occurrence of pains resembling those of labour at the end of the 9th month, affords a presumption in favour of conception having taken place, which did not exist in the case reported by Dr. Grossi. It seems somewhat doubtful whether the case of Dr. Yardley's patient was one of extra-uterine pregnancy, or of rupture of the womb towards the end of gestation, from external violence, with escape of the fœtus into the abdominal cavity. After 6 months of severe suffering, the patient regained her health; she gave birth to a dead child four years afterwards, and subsequently miscarried thrice. During almost the whole of this time, the woman's health was indifferent, and her sufferings were severe, till at the end of 15 years the fœtal bones made their escape through the rectum, after which, in the course of the ensuing five months, she completely recovered. The account given by Dr. Craddock, refers to the dissection of a woman, in whose abdomen an extra-uterine fœtus had resided for 22 years without impairing her health; death taking place eventually from pneumonia. Dr. Carganico's patient died of hemorrhage, produced by rupture of the cyst at 5½ months. The anatomical details are very incomplete, but it is stated positively that the placenta was attached to the peritoneum in the pouch between the uterus and rectum, and not to the ovary. Dr. Mason's patient died exhausted, 4 months after the natural end of pregnancy; a communication having formed between the cyst and intestines, as well as an opening through the abdominal walls. The woman, whose history is recorded by M. Cerise, died after 15 hours of pain like that of labour, occurring at the full term of pregnancy. The anatomical details of the case are very imperfectly given, and the insertion of the placenta is not stated. In the cases of Drs. McCulloch, Whinery, Stevens, and Götz, gastrotomy was performed. More than 18 years had elapsed since the natural termination of pregnancy in the first case, more than 4 years in the second, more than 10 years in the third, but in the fourth case, the operation was performed at the end of 9 months, and a child was extracted, which survived for two hours. The placenta was large, and so firmly attached to the fundus uteri, that it was thought imprudent to attempt separating it. The patient went on well for a few days, but the placenta becoming partially detached, fatal hemorrhage took place on the eighth day after the operation. The insertion of the placenta was found to have been to the right ovary, and the right side of the fundus uteri. [These cases substantiate the general opinion that gastrotomy, at the natural end of pregnancy, is attended with great peril, while if restricted to patients in whom nature is, after the lapse of some time, endeavouring to get rid of the fœtus through the abdominal parietes, a successful issue may often be expected.]

### NATURAL LABOUR.

Dr. Simpson* expresses the opinion that *galvanism does not exert any influence, either in originating or in increasing uterine action.* The experiments which led him to this conclusion were made on six women, in whom he carefully noticed the duration of the labour-pains, and of the intervals between their recurrence. He next repeated his observations with all the apparatus for galvanism prepared, but without establishing the contact, he then established contact, and lastly, renewed his observations after removal of the wires. From these experiments he infers that when uterine action has seemed

* Monthly Journal, July, 1845.

to be excited by means of galvanism, this has either been a mere coincidence, or has resulted from the impression made on the mind, or been produced by the mechanical irritation of the os uteri, or of the surface of the abdomen by the conductors. [These observations appear to have been made with great care, but can hardly as yet be allowed to outweigh the results arrived at by Reil and Carus in their experiments upon animals, and the recent evidence in favour of the reality of the influence of galvanism afforded by some of the cases which Dr. Radford has recorded.]

*Plurality of Birth.* Mr. Pretty* relates a case in which a woman gave birth in the 7th month of pregnancy to three male children, of whom only one was living. Cases in which four children were born in one labour are recorded by Dr. Beatty†, and Dr. Migliavacca‡. Three of the children in Dr. Beatty's case were born alive; one, an acephalous monster, was still-born. In the other case, the sex of the children is stated; three were girls, the fourth was a boy. They were all born alive, but all died speedily, the boy, who survived the longest, living only nine days.

PRETERNATURAL LABOUR, FROM CAUSES DEPENDING ON THE MOTHER.

*Malformation of the Pelvis.* Dr. Kirchoffer§ has described the case of a young woman who was delivered of her first child by the Cæsarean section, in consequence of the want of capacity of her pelvis, and died of hemorrhage five days afterwards. Her pelvis (of which a very interesting cast has been published) was found to be perfectly healthy, but presenting a remarkable similarity to the quadrilateral pelvis described by Robert of Marburg, of which mention is made in the first of these Reports. Like it there is defective development of the alæ of the sacrum, and anchylosis of both sacro-iliac synchondroses.

| | |
|---|---|
| The distance between the two anterior superior spines of the ilium was only | 7 inches |
| Antero-posterior diameter of the brim | 4½ „ |
| Transverse | 3 „ |
| Antero-posterior diameter of the outlets | 4 „ |
| Transverse | 1 „ |

Dr. Kirchoffer subscribes to Naegele's theory of the congenital origin of this malformation, an opinion which is supported by Dr. Moleschott‖, in a well-written essay on the subject, though he does not adduce any new facts or arguments in its support.

Professor Martin, of Jena,¶ has revived the arguments on which he insisted some years ago, in order to prove this deformity not to be congenital. The history of a case recorded by Danyau, in which hip-joint disease, that had terminated by anchylosis of the head of the femur into the acetabulum, existed on the contracted side of the pelvis, is regarded by him as affording a confirmation of his views. The left hip-joint had been diseased, the left sacro-iliac synchondrosis was completely ossified, the left half of the sacrum was narrower than the right, as was also the left ramus of the pubes, and the whole of the left os innominatum was less perfectly developed than the right. The left oblique diameter of the pelvic brim was 4 inches, 4 lines, and the right, 3 inches, 10 lines. [This case might be regarded as affording support to Martin's theory, that the deformity is the result of an ulcerative process, in the course of which the bone becomes absorbed, and the disease finally cured by anchylosis of the joint, if we did not know that Vrolik's researches** have proved the effect of

* Med. Gazette, Dec. 5, 1845.
† Dublin Med. Press, Dec. 10, 1845.
‡ Gaz. Med. di Milano, May 9, 1846.
§ Neue Zeitschrift f. Geburtsk., xix, 3tes Heft.
‖ Zeitschrift für die gesammte Medicin, April, 1845.
¶ Neue Zeitschr. f. Geburtsk., xix, 18tes Heft.
** Essai sur les effets produits dans le corps humain par la luxation congénitale, etc., du Fémur. 4to. Amsterdam, 1839.

hip-joint disease to be a widening rather than a contraction of the affected side of the pelvis. A preparation in the museum of the Middlesex Hospital bears witness to the correctness of Vrolik's views, as probably do specimens in other anatomical collections. We must then conclude that the combination was in this case purely accidental.]

Dr. David and Mr. Gibson* have each related a case in which the *pelvis* was *narrowed by fracture of the sacrum.* In Dr. David's patient the accident took place in the fourth month of her fourth pregnancy, notwithstanding which she went to her full time, but delivery was then found to be impracticable, and consent not being given by her to the performance of craniotomy, she died undelivered. Mr. Gibson's patient was twice delivered by craniotomy after the occurrence of the accident, [but no reason is assigned for the induction of premature labour, with the view of saving the children, not having been attempted.]

*Morbid states of the uterus.* Cases of the *successful incision of the os uteri* in persons whose delivery was rendered impracticable by its occlusion, are related by M. Laborie, Mr. Davis, and Dr. Y. Ona.† One of M. Laborie's cases is of importance, since it illustrates an occasional source of danger from the operation; hemorrhage so profuse having followed the incisions as to place the patient's life in danger.

Dr. Lever‡ relates a very interesting case of *separation of the lower segment of the uterus* in a case where labour was obstructed by insuperable rigidity of the os uteri. The patient died on the 11th day after the occurrence, puerperal affection of the joints having come on the day following her delivery.

M. Devilliers§ has related the history of a lady who conceived after five years of childless marriage; an impediment to conception having existed in a *congenital constriction of the vagina,* which narrowed the passage so considerably as scarcely to allow the finger to pass beyond it. When labour came on, however, no interference was needed, the constriction yielding readily, and allowing parturition to be accomplished without difficulty. From this fact, he deduces the inference [which many cases already on record would substantiate] that congenital narrowing of the vagina may almost always be left to nature, although the acquired narrowing which results from inflammation and other similar causes generally needs interference.

Dr. W. Lange‖ mentions an instance of *persistence of the hymen,* rendering its division necessary during labour. [It seems likely, however, from the situation of the membrane, 1½ inch from the vulva, that it was not the hymen, but a secondary membrane situated beyond it.]

M. Danyau¶ describes a case in which labour was complicated by the presence of a large fibrous polypus connected with the anterior lip of the uterus, and which was forced by the labour-pains beyond the vulva. The woman had been delivered by a midwife, who turned the child before M. Danyau saw her; he, however, divided the pedicle of the tumour, which was two fingers' breadth in diameter, and no serious symptom followed the operation. In some observations appended to the case, he points out the danger of inducing abortion by meddling with such growths before pregnancy is terminated, but he does not think it necessary any longer to postpone operating after labour has taken place.

*Inversion of the uterus.* Dr. Fisher** describes a case of the spontaneous inversion of the uterus during labour at the 8th month; the fœtus and pla-

* Lancet, May 16, 1846; ibid., June 13, 1846.
† Gaz. Méd., Jan. 31, 1846; Med. Gazette, March 20, 1846; Amer. J. of Med. Science, Oct., 1845.
‡ Guy's Hospital Reports, 1845. § Revue Médicale, Août, 1845.
‖ Oesterr. Med. Wochenschr., Oct. 4, 1845.
¶ Gaz. Méd., Oct. 3, 1846, from Journal de Chirurgie, Juin, 1846.
** American Journal of Med. Science, May, 1846.

centa having been expelled together at at the moment when the accident happened. The cord, in this instance, was only eight inches long, and in a case related by Mr. Smith,* its length did not exceed six inches. The placenta was, in this instance, found partially detached from the inverted womb, immediately after the birth of the child, but after its complete separation the womb was returned to its proper position with but little difficulty. The uterus was likewise easily reduced in Dr. Fisher's case, and in neither was the accident attended by serious hemorrhage. A third case is related by Dr. Christie,† in which the patient died of hemorrhage, the uterus having been inverted, and the placenta in part detached as the result of traction at the funis by an ignorant midwife.

The note‡ contains references to several fatal cases of *rupture of the uterus.* The history of Dr. Hersing's patient strikingly illustrates the bad results of neglecting enbryotomy, and of trying to turn at all hazards, when the liquor amnii had long escaped, the uterus was acting spasmodically, and when, moreover, the prolapsed umbilical cord was felt to be pulseless. The accident occurred to Dr. Bona's patient, during an attempt to drag a child by means of the forceps through a pelvis with a conjugate diameter of two inches. Mr. Tyte, after allowing fifteen hours to elapse from the occurrence of symptoms of rupture of the uterus before he interfered at all, then applied the vectis, and afterwards the forceps, and finally delivered by craniotomy. In Dr. Reid's patient the accident occurred in the course of a natural labour, which had not lasted above 4 hours, the liquor amnii having escaped only half an hour, and the os uteri being dilatable. The case is interesting, moreover, from the absence of those symptoms of great nervous depression which usually come on immediately after the occurrence of laceration. The patient died in 36 hours, with symptoms of uterine inflammation, which had appeared to call for active treatment. Mr. Jackson's patient had been for 12 hours in her 4th labour, her former labours having been tedious, owing to slight contraction of the pelvis, when the uterus gave way, and the child escaped into the abdominal cavity. Gastrotomy was performed about three hours after the occurrence of the accident, and a dead female child extracted. The patient went on tolerably well for the first three days, when symptoms of extreme exhaustion came on, and on the 8th day she died. From a comparison of this case with thirteen others that came under his notice, Mr. Jackson draws a conclusion favorable to gastrotomy, whenever a considerable time has elapsed since the escape of the fœtus into the abdominal cavity; for in those cases in which either turning was performed, or the patient was left to nature, death always took place within three days, while the woman on whom gastrotomy was performed survived for 8 days.

Two instances of recovery after rupture of the uterus are recorded; the one by Dr. Williamson, the other by M. Robiquet.§ In the first case the feet of the child presented, one of which, during a violent pain, perforated the lower segment of the uterus, about an inch from the edge of the os. Immediate suppression of the uterine action followed the accident, but the foot being returned, labour was completed by the natural powers. The absence of bad symptoms in this case might be in some measure accounted for by the comparative slightness of the injury. In M. Robiquet's patient, the uterus gave way spontaneously after about 24 hours of tedious but not severe labour, and no very grave symptoms succeeded the occurrence. After the delivery of the child, which was effected by the forceps, a portion of omentum and a coil of small intestine prolapsed at the vulva. Soon after their return, however, the

* Monthly Journal, May, 1846. † Ibid.

‡ Hersing, Med. Zeitung, 30 Juli, 1845; Bona, ibid., Nov. 12, 1845; Tyte, Lancet, Feb. 7, 1846; Reid, Med. Gazette, Aug. 15, 1845; Jackson, Prov. Med. Journal, Sept. 3, 1845.

§ Northern Journal, Sept., 1845; Bull. de l'Acad. de Médecine, April 30, 1846.

uterus contracted well, no bad symptom supervened, and on the 17th day the patient was quite convalescent.

In a clinical lecture, by M. Roux,* on *lacerated perineum,* that surgeon expresses himself as decidedly in favour of operating some considerable time after delivery, rather than immediately or shortly afterwards. He considers the operation at the late period to be attended with much less danger of the supervention of erysipelas, and more likely to lead to the complete closure of the wound. In 13 out of the 15 cases on which he operated, his success has been complete, but in the remaining two cases the patients died. M. Roux employs the quilled suture, having previously pared the edges of the wound, and inserts only three stitches. For the first few days he keeps a catheter in the bladder, but does not consider it necessary to maintain a constipated state of the bowels after the 6th day, and so soon as they have acted freely he cuts out the stitches. The consolidation is quite firm, and there does not appear to be any increased tendency to laceration in a subsequent labour, but rather the reverse.

#### LABOUR PRETERNATURAL, FROM CAUSES DEPENDING ON THE CHILD.

Of this but few cases have been related. Two instances are recorded,† in which the *spontaneous evolution* of a second twin took place; the child in the former case being still-born, in the latter born alive.

Mr. Elton‡ has described a case of twin labour, in which the birth of the first child, which presented by the feet, was prevented by its head and that of the second child becoming locked together, just as in the case reported by Dr. Walter, and mentioned in the last Report. Mr. Elton decapitated the first child, and then extracted the second by the forceps, the liquor amnii having escaped so long before that he feared making any attempt to disengage the two heads. The second child was still-born as well as the first.

#### OPERATIVE MIDWIFERY.

Dr. Hoffman's report of the lying-in hospital at Würzburg§ is of much value as an unintentional illustration of the dangers of that over-fondness for manual interference that characterizes many of the continental practitioners, and especially of the hazard attending the *injudicious employment of the long forceps.* Dr. Hoffman applied the forceps 20 times in 637 labours, or about once in 30 cases. Dr. Ramsbotham once in 729 cases. The deaths after application of the forceps in Dr. Hoffman's practice were 35 per cent.; in Dr. Ramsbotham's practice 6·12 per cent. As illustrative of the dangerous force that may be exerted by this instrument, the case may be mentioned in which Dr. Hoffman having applied it while the head was at the brim of the pelvis, succeeded in extracting the child, but in so doing tore asunder all the joints of the pelvis, and lacerated the urethra and vagina, of which injuries the woman died on the 18th day. In a second case, the application of the forceps to extract a putrid child was followed by the mother's death in 29 hours. In another instance where the cord was prolapsed and pulseless, a dead child was extracted by the forceps, and the mother died 4 days afterwards of puerperal fever. In a fourth case, the long forceps was applied to draw a hydrocephalic head into the pelvis, and traction was made with the blades unlocked; though on a reapplication of the instrument, some time afterwards, it was found possible to lock it. Craniotomy was at length performed, but the woman died 24 hours afterwards, her vagina being extensively lacerated.

*Craniotomy.* M. Reali || has related another instance of that horrible oc-

* Gaz. des Hôpitaux, Oct. 7, 1845.

† Hersing, Med. Zeitung, Dec. 3, 1845; Dr. Reid, Med. Gazette, Aug. 15, 1845.

‡ Med. Gazette, July 24, 1846

§ Neue Zeitschr. f. Geburtsk., xx, 1stes und 2tes Heft.

|| Gaz. Méd., Sept. 27, 1845.

currence, the survival of a child for two hours after it had been extracted by means of Levret's *tire tête,* and quite a fourth of the brain had escaped during the operation. [It must be borne in mind that the instrument employed is a branched crotchet, which is applied without previous perforation of the skull.]

*Cæsarean section.** Four cases are related of the performance of this operation, with a favorable result both to mother and to child; 2 in which the mother survived, 6 in which the life of the child was preserved, and 2 in which neither life was saved. The history of the mother, in Mr. Goodman's case is not carried beyond the third week, at which time, however, she was doing well. In the patient on whom Dr. Meyer operated, the uterus contracted around the neck of the child so firmly after the body was extracted, as to render it necessary to enlarge the incision, an accident which illustrates the advantage of extracting the head first whenever that is possible. Mr. Lyon's case presents many points of interest. The operation was rendered necessary by the presence of a tumour blocking up the pelvis, and which, from its position behind the rectum, as well as from its firmness, was taken for an osteosteatomatous tumour of the pelvis. It turned out, however, to be the left ovary, enlarged and converted for the most part into an adipocire-like substance. [The case derives great importance from being almost, if not quite, the only instance of an ovarian tumour getting behind the rectum, and it illustrates the necessity of making an experimental puncture or incision of such tumours through the vagina, before exposing a patient to the dangers of the Cæsarean section.] In the case reported by Mr. Aitken, the uterus had given way before the patient's admission into the hospital, so that the Cæsarean section, which the extreme contraction of the pelvis rendered necessary, could not be regarded as the sole cause of her death. [The statistics of the operation at present yield the following results. It has been performed in 378 cases, of which trustworthy accounts have been given. In 145 of these cases the women recovered, in 233 they died; or the recoveries were in the proportion of 38 per cent., or as one in 2·6 cases. The fate of 318 children is mentioned, of whom 219 were saved, 99 were lost, or the child survived in 68 per cent., or in rather more than 2 cases out of 3.]

### UTERINE HEMORRHAGE.

Professor Simpson† has published some remarks on the anatomical source and pathological nature of post-partum hemorrhage, which have a direct and very obvious bearing on the opinion he has expressed with reference to the source of the hemorrhage in placenta prævia, and the treatment applicable to some cases of it. He notices the fact that hemorrhage sometimes takes place after delivery, notwithstanding the existence of an average amount of uterine contraction, while, on the other hand, hemorrhage does not always follow the expulsion of the placenta, though the uterus is imperfectly contracted. Bleeding is prevented, not merely by the degree, but also by the equability and uniformity of the uterine contraction, while other means besides muscular contraction concur in producing the same effects. Hemorrhage from the detachment of the placenta is never arterial, but always takes place from the veins; the blood that ought to flow onwards towards the periphery of the

* Both lives saved—Dittmar, Gaz. Méd. de Strasbourg, and Dublin Journal, Nov., 1845; Long, Gaz. Méd., Sept. 13, 1845; Künsemüller, Neue Zeitschr. f. Geburtsk., xix, p. 384; Steinbrenner, Gaz. des Hôpitaux, Sept. 12, 1846. Mother survived—Goodman, Med. Gaz., Dec. 26, 1845; Meyer, Med. Zeitung, Sept. 10, 17, 1845. Child survived—Lyon, Monthly Journal, Dec., 1845; Jungmann, two cases, Oesterr. Med. Jahrb., Sept., 1845; Kirchoffer, Neue Zeitschr. f. Geburtsk., xix, 3tes Heft; Balfour, Northern Journal, May, 1846; Aitken, Lancet, June 13, 1846. The last two cases occurred at Vienna, and are merely reported by Messrs. Balfour and Aitken. Neither life saved—Künsemüller, loc. cit.

† Northern Journal, January, 1846.

uterus, and the ovarian and hypogastric trunks, regurgitating towards the cavity of the organ. Bleeding from the lacerated openings in the veins is checked as well by a peculiarity in the structure and arrangement of these vessels as by the constriction of their orifices by the uterine fibres. This peculiarity, noticed by Mr. Owen, in vol. iv, of the collected edition of Hunter's Works, and more recently by Mr. Goodsir, consists in the arrangement of the veins in successive tiers or planes, the veins of the lower tier communicating with those of the upper, by very oblique openings in the floor of the latter, while the opening into the lower vein is partially covered by a semilunar projection, formed by the lining membrane of the two venous tubes as they meet together at a very acute angle. It is probable, moreover, that these semilunar processes act something in the manner of the Eustachian valve, and thus further contribute to prevent the regurgitation of blood from above downwards. Another circumstance, which is not without its influence in diminishing the tendency to hemorrhage after the expulsion of the placenta, is that the derivative power which attracted the blood downwards into the placental cells no longer exists; and this having ceased, the blood will be more likely to flow in the onward current, and by direct channels, than to pass through the less free communications which exist between two different tiers of veins. The formation of coagula in the collapsed veins, and the presence of tufts of fœtal vessels, or remnants of decidua blocking up their openings, likewise contribute to the same result. It is obvious that these facts, assuming them to be correctly stated, lend much support to Dr. Simpson's opinion, which was also entertained by the late Dr. Hamilton, that the main bleeding in cases of placenta prævia takes place not from the uterine surface, but from the placental orifices of the lacerated veins, from which the blood would escape in its natural onward course, unchecked by any contractile power in the veins themselves, or in the tissue by which they are surrounded.

Dr. Radford,* Dr. Ashwell,† and an anonymous correspondent of the 'Medical Gazette,'‡ writing from Glasgow, under the signature of J. B., attack this theory of the source of the hemorrhage in placenta prævia as being physiologically impossible. Whether correct or not, however, this theory involves no contradiction of ascertained anatomical facts; its alleged incompatibility with the account given by Weber, Wagner, and others, evidently rests on a misunderstanding of Dr. Simpson's statement.

The pamphlet of M. Négrier,§ though written with the express object of explaining the causes of presentation of the placenta, and of laying down rules for the management of cases of hemorrhage from that source, contains little either new or valuable. The supposition that the placenta becomes attached to the cervix uteri, in consequence of the ovum having entered the womb while the decidua was as yet unformed, and having consequently gravitated to the lowest part of its cavity, has often been entertained, though now regarded as an inadequate, probably, an altogether incorrect explanation of the occurrence. The fact, too, that the fibres of the cervix are differently arranged from those of the body of the uterus, and that the cervix is likewise possessed of a smaller amount of contractile power, have both been long known, although brought forward as novelties by M. Négrier.

The papers on the subject of placenta prævia, by Drs. Simpson and Lee,‖ are occupied chiefly with the exposure of statistical errors into which each conceives the other to have fallen, and leave the main question of the propriety of detaching the placenta before the birth of the child, much as it stood last year. The interest of the subject, however, has led to the publi-

* Med. Gazette, Nov. 14, 1845. † Ibid., Nov. 7, 1845. ‡ Ibid., Nov. 21, 1845.

§ Recherches et Considérations sur la Constitution, et les Fonctions du Col de l'Uterus, &c. 8vo, Paris et Angers, 1846.

‖ Med. Gazette, Sept. 19, Oct. 10, 24, and Nov. 7, 1846.

cation of cases in which the placenta was spontaneously expelled before the child, as well as to its artificial detachment in various instances, where the experiment was conceived to be justifiable. Eleven cases are mentioned,* in which the spontaneous expulsion of the placenta occurred, the accident having happened twice to the same woman. All the mothers survived, except one, who died of the effects of the loss of blood on the 8th day. Nine of the children were still-born, one was very feeble at birth and died in a few hours, and one survived. The interval between the expulsion of the placenta and the birth of the child is stated in 7 cases. Thrice the interval did not exceed a very few minutes, once it was ten minutes, once half an hour, and once three hours. The hemorrhage appears to have ceased in every instance on the expulsion of the placenta, but in both instances in which the child was born alive its birth followed almost immediately on the expulsion of the placenta.

Seventeen instances have been recorded in the English Journals† during the past fifteen months, of detachment of the placenta before the birth of the child, in cases of placenta prævia. In the case recorded by Dr. Simpson, to whom it had been communicated by Mr. Cripps, the placenta was removed by an ignorant midwife, and 10 hours elapsed before the child was born, during which time, however, no hemorrhage took place. In 16 out of the 17 cases the bleeding is said to have ceased immediately on the detachment of the placenta, but Dr. Everitt mentions that although the flooding abated on the separation of the placenta, it did not entirely cease until after the application of cold externally; and he insists on the fact as proving that in cases of this kind, the hemorrhage comes from the uterine as well as the placental ends of the lacerated veins. The life of the mother was preserved in every case but one, and then the previous hemorrhage had been so profuse as almost to exhaust the patient, who died 3 hours after delivery. All the children were still-born, except in the case related by Mr. Stickings. [As far as the well-doing of the mother is concerned, the results of these cases must be regarded as favorable; but, on the other hand, the lives of 17 out of 18 children were sacrificed, at least half of whom would probably have been saved by the ordinary practice. In many instances, too, there appears to have been no reason why the child was not turned and extracted first; the os uteri having been well dilated, or yielding and dilatable. In such cases it seems not unfair to assert that the child's life was sacrificed to the desire of performing a new operation. Several of the cases are so loosely worded that little can be gathered from them, while some have either been so carelessly observed, or so incorrectly related as to render them quite untrustworthy.]

The opinion expressed by some practitioners, and acted on by more, that the new mode of treatment is generally applicable, and to be preferred to the ordinary practice, is opposed both by Mr. Crowfoot‡ and Dr. Radford,§ the latter of whom disclaims having recommended the detachment of the placenta before the birth of the child except under special conditions, these being the death of the child, the existence of so great a degree of exhaustion from loss of blood, as to render the ordinary mode of proceeding impracticable, or

* Goddard, Lancet, Dec. 3, 1845; Parker, Prov. Med. Journ., Sept. 24, 1845; Favell, ibid., Aug. 20, 1845; Reid, Med. Gazette, Nov. 25, 1845; Tweed, Lancet, Jan. 3, 1846; Ley, Prov. Med Journal, April 22, 1846; Russell, Ed. Med. Surg. Journal, July, 1846, p. 52; and Burwell, Amer. Journ. of Med. Science, April, 1846.

† Wilkinson, Prov. Med. Journal. July 23, 1845; Walker, ibid., Sept. 3; Greenhow, ibid., Sept. 10, 1845; Maclean, Northern Journal, Aug., 1845; Radford, Med. Gaz., Oct. 24, 1845; Jones, Lancet, Sept. 27, 1845; Wells, ibid., Nov. 8; Brown, ibid., Dec. 27, 1845; Simpson, Med. Gazette, Oct. 10, 1845, p 1011, note; Hutchinson, Prov. Med. Journal, Oct. 15, 1845; Houghton, Lancet, Jan. 24, 1846; Stickings, Med. Gazette, May 8, 1846; Wales, Prov. Med. Journal, April 8, 1846; Bryan, ibid., June 17, 1846; Jay, Med. Gazette, Aug. 21, 1846; Everitt, Prov Med. Journal, Sept. 30, 1846.

‡ Prov. Med. Journal, Nov. 12, 1845. § Ibid., Aug 13, 26; and Med. Gaz., Nov. 21, 1845.

the presence of some mechanical obstacle to the extraction of the child. He further condemns the employment of a sound, or of any instrument for detaching the placenta, and urges that the operation should be undertaken only when it can be accomplished by the finger, and when the introduction of the hand into the uterus has become practicable. Previous to this, the application of cold, but above all, the employment of the plug must not be omitted. These limitations would reduce the cases suited for the new plan of treatment to a very small number, but it must be borne in mind that Dr. Simpson* himself is very far from recommending it as always applicable, but confines it to those cases where rupturing the membranes is insufficient, and where turning is either impracticable or extremely dangerous. The cases in which he would practise it, are those in which the os uteri is rigid and undilated, the uterus too contracted, or the woman too exhausted for turning to be safely performed, or where the pelvis is contracted. Further, he would adopt it when the child is premature, or has been ascertained to be dead, and also in the greater number of cases of first labour, or of labour coming on before the 7th month.

Professor Osiander, of Göttingen,† is the only continental writer who has noticed this question, and he objects most strongly to the practice of detaching the placenta before the child, on the ground of its necessarily involving the death of the child, while he doubts, though apparently not on the ground of facts observed by himself, whether the practice be a certain means of arresting hemorrhage.

Many isolated cases are related of the successful treatment of placenta prævia by turning the child. References are not made to them in this Report, because the mere fact of that plan being adequate in the majority of instances is generally admitted, while nothing but a most elaborate analysis of cases of both kinds would render any inferences as to the comparative value of the two modes of treatment, drawn from their statistics, at all trustworthy. This is well illustrated by a statement of Mr. Russell's,‡ that he saved all the mothers, and 6 of the children, in 7 cases of placenta prævia that occurred in his private practice, while he lost 5 women out of 29 to whom he was called in consultation. The different results here depended not on the treatment, but on the time of its adoption.

Mr. Dorrington§ has related a case of placenta presentation in which he employed galvanism with apparent advantage, but the patient had not presented any very alarming symptoms, and galvanism was not employed alone, but in conjunction with rupture of the membranes, so that it is not easy to determine to which of these measures the excitement of vigorous uterine action, and consequent arrest of the hemorrhage, are to be attributed.

Dr. J. H. Davis|| animadverts on Dr. Simpson's omission of all mention of the plug, as a means to be employed in the management of cases of placenta prævia, and insists on its extreme value in controlling hemorrhage until the state of the os uteri is such as to allow of turning. M. Négrier,¶ likewise, extols the plug, but at the same time he absolutely condemns the rupture of the membranes, since the cervix uteri being possessed of but little contractile power would not, in his opinion, close the bleeding vessels. In cases of central insertion of the placenta, he advises that the hand be introduced through it rather than to one side, since, by the former plan, the mother will lose less blood, while the fœtus will generally be born alive, if delivery be expeditiously accomplished. He recommends, moreover, the forcible dilatation of the os uteri, in all cases where women have previously given birth to children, since he thinks that in them it seldom offers any considerable resistance. [M.

* Med. Gaz., Oct. 10, 1845, p 1013.

† Neue Zeitschr. f. Geburtskunde, xix, 3tes Heft.

§ Prov. Med. Journal, March 11, 1846.

|| Lancet, Nov. 8, 1845.

¶ Lib. cit., p. 133-170.

Négrier's own results, (he lost 4 patients out of 8) form the best comment on doctrines, which, but for the reputation of the author, would not have been noticed.]

A controversy has been carried on in Italy, which does not seem to have attracted much attention in other parts of the continent, between Dr. Bellini, of Florence, and some of his countrymen, with reference to the treatment of placenta prævia.* Dr. Bellini advocates the making incisions into the os uteri, in cases of hemorrhage from this source, in order to allow of the early introduction of the hand, and consequent early delivery of the patient. He condemns the use of the plug in these cases as being a means either wholly inefficient, or at best, suppressing the bleeding only for a short time, while it favours the occurrence of internal hemorrhage. On the other hand, incisions have often been made into the os uteri for the express purpose of enabling the practitioner to deliver his patient immediately, and no bad results have followed from this proceeding, even in cases where the use of the forceps has been needed to accomplish delivery. The four instances in which Dr. Bellini resorted to incision of the os uteri were not cases of placenta prævia, and it has accordingly been objected by Ciniselli, that the state of the os uteri, when the placenta is attached around it, differs from its condition in ordinary labour, it being thick and vascular, and consequently likely to bleed dangerously if incised. The other arguments for and against incision of the os uteri, and in favour or in dispraise of the plug present nothing remarkable.

Mr. Dorrington† relates two cases, and Dr. Radford‡ one, of the successful employment of galvanism to excite uterine action in accidental hemorrhage. In each instance the agent seems really to have had the effect attributed to it; but Dr. Radford's case is the most conclusive, since rupture of the membranes had been previously resorted to without the uterine action being in the least degree excited by it. Another case is recorded by Mr. Johnson§ of its employment to check dangerous hemorrhage from the uterus, a month after miscarriage.

A very interesting case is related by Dr. Pagan,|| in which hemorrhage, after the expulsion of the placenta, appears to have been kept up by that body having been partially developed within the fallopian tube. The placenta was disrupted, and the hand, when introduced into the uterus, removed a portion, 2 inches long by half an inch broad, and expanding into a surface 2½ inches in breadth from one side of the fundus of the organ. He supports his opinion as to this deviation from the natural seat of the placenta being an occasional cause of flooding, by the detail of another case in which the patient died of peritonitis, she having had hemorrhage after delivery, for which the placenta was extracted, though lacerated in so doing. After death a portion of placenta, 3½ inches long, was found at the orifice of the tube, attached around its margin, and projecting far into its cavity. Some similar observations have been made by Riecke and d'Outrepont, to which Dr. Pagan refers.

Mr. Adams¶ has written some essays on floodings after delivery, and their treatment, which are remarkable for their opposition to all hitherto received opinions on the subject. He asserts that in the majority of cases hemorrhage after delivery does not result from want of uterine contraction; that the blood does not proceed from the interior of the uterus at all, but from the rupture of vessels about the os tincæ, or more frequently about the vulva, during the passage of the child.

* An account of part of this controversy is given by Schreiber, Neue Zeitschr. f. Geburtsk., xvii, 2tes Heft; besides which there are, an essay by Bellini, in Gaz. Med. di Milano, Nov. 15, 1845, and a defence of the use of the plug by Barbieri, in the same number of the journal; and by Casazza, ibid., Aug. 15, 1846.

† Prov. Med. Journal, March 11, 18, 1846.

‡ Med. Gazette, Jan. 2, 1846.

§ Prov. Med. Journal, March 25, 1846.

|| Monthly Journal, Nov., 1845.

¶ Med. Gazette, Aug. 29, 1845, Jan. 23, 1846.

He concludes, therefore, that the introduction of the hand into the uterus is not only useless, but mischievous; that the local application of cold, gentle compression of the abdomen, and the administration of small doses of opium are usually all that is needed; and that if these means should fail, the bleeding vessel at the vulva must be sought for, and secured by ligature.

These extraordinary assertions are refuted by Dr. Ramsbotham* and Mr. Copeman,† who ably vindicate the usual mode of practice.

Dr. Beattie‡ has made some remarks on the value of the ergot of rye as a means of preventing hemorrhage after delivery, in cases where there may be reason to dread its occurrence. With this view, in cases where hemorrhage has followed the expulsion of the placenta in previous labours, or where the incomplete contraction of the uterus causes him to dread its coming on, he administers a dose of ergot immediately on the birth of the child. He conceives that the remedy acts both by lowering the action of the heart, as well as by inducing contraction of the uterus. He mentions, moreover, that he has found it of great service when administered in this way, as a means of preventing those intensely severe after-pains which some women suffer, and which opium fails to relieve. He conceives that in these cases it acts by producing a complete contraction of the uterine fibres, and thus preventing the formation of clots in the interior of the organ, by which spasmodic action is excited and kept up.

M. Négrier§ is one of a small number of practitioners who place confidence in the plug, as a means of arresting hemorrhage after delivery. He regards it as very useful in exciting uterine action, while he conceives that by pressure upon the womb, in the direction from above downwards, the danger of internal hemorrhage may be avoided. Two cases of its successful employment after delivery are related by Dr. Barbieri,|| but in one of these cases 10, and in the other, 9 days had intervened between the woman's confinement and the commencement of flooding.

Mr. Pretty¶ suggests a modification of the pad and tourniquet, which have been used by different practitioners to suppress uterine hemorrhage after delivery. This modification consists in the introduction of two lateral pads to compress the sides of the uterus, in addition to the larger one, which presses it from before backwards.

Mr. Brown** has related a case where, exhaustion having come on after craniotomy, although it does not appear that the quantity of blood lost was very considerable, he performed transfusion, and succeeded in restoring the patient by the injection of ℥iv of blood.

### THE PUERPERAL STATE.

*Puerperal Convulsions.* Dr. Mickschick,†† to whose investigations on the subject of kysteine reference has already been made, tested the urine of 26 women in labour, with the view of ascertaining the correctness of Lever's statements as to the presence of albumen in the urine in cases of convulsions. He detected it in the urine of 5 patients, one of whom was dropsical. Two women were attacked with convulsions, but albumen was found in the urine of only one of them. M. Labat‡‡ has reported an interesting case where puerperal convulsions came on in a highly anasarcous woman, who at length sank into a comatose condition. By degrees, as the anasarca diminished under the influence of remedies, the albumen, with which the urine had been

* Med. Gazette, Sept. 12, Oct. 24, 1845.
† Ibid., Nov. 7, 1845.
‡ Dublin Quarterly Journal, May, 1846.
§ Lib. cit., pp. 152-171.
|| Gazetta Medica di Milano, Nov. 15, 1845.
¶ Med. Gazette, Jan. 16, 1846.
** Northern Journal, Dec., 1845.
†† Loc. cit.
‡‡ Gaz. des Hôpitaux, May 30, 1846.

loaded, disappeared, and the intellectual powers returned. Dr. Landsberg* has published a very prolix essay on the subject of puerperal convulsions, the chief aim of which is to point out that the danger attending them arises, not from the convulsions, but from the accompanying congestion of the brain, and that free depletion must, therefore, always be practised, while forcible delivery is not only useless, but dangerous.

*Puerperal Fever.* MM. Bidault and Arnault have given an account† of a very fatal epidemic of puerperal fever, which they observed among the lying-in patients at the Hôtel Dieu and Hôpital St. Louis, in the years 1843-4. Its greatest prevalence was during the colder months of the year, namely, from September to March, and its fatality was such, that of 34 who were attacked, only 2 recovered; and these 34 cases occurred among 156 women. The general characters of the disease, were those of the epidemic gastric fever of Locock. The veins of the uterus were unaffected, but its lining appeared, covered with a gray sanious false membrane; the uterine lymphatics, especially near the appendages of the organ, were full of pus and peritonitis, or purulent effusion into the peritoneum was present in every case. Among those who died at the Hôtel Dieu, there was likewise found an enlarged and diseased condition of Brunner's glands. The treatment consisted in the employment of depletion and mercurials; and the empirical administration of the tincture of aconite in some cases, among which were the two that recovered. From an editorial article in the 'Gaz. Médicale' of Feb. 28, 1846, the disease seems to have been extensively prevalent during the past winter, especially among the inmates of the hospitals. The atmosphere of the hospitals appears to have had a great share in producing it, for at the time when it was most prevalent, many women who were received into those institutions for various diseases, and who had been delivered some months previously, or who were menstruating, were attacked by inflammatory affections of the generative system. During the autumn of 1842, and till late in the spring of 1843, puerperal fever was very prevalent in Würzburg and its vicinity, and attacked 30 of 129 women who were delivered during that period in the lying-in hospital of the city, 14 of whom died. The uterine appendages, the peritoneum, and the membranes of the chest and head, were the parts most frequently affected, while the substance of the uterus was seldom implicated. The course of the disease was generally extremely rapid, and patients were attacked by it after the easiest labours, as often as after labours that had been tedious or difficult. Premature labour occurred in many cases, as though the seeds of the disease had been sown in pregnancy; a supposition which seems the more probable since uterine hemorrhage was likewise of frequent occurrence, and was often followed speedily by the outbreak of the fever. Although apparently originating under atmospheric influence, and appearing simultaneously in different parts of the city, there were yet many instances in which it was fairly traceable to contagion.‡ The contagious nature of puerperal fever, forms the subject of an elaborate compilation by Dr. Kneeland,§ and in a second similar communication, he endeavours to illustrate the connexion between it and epidemic erysipelas, which he believes to be produced by the same morbid poison. This supposition is supported by the interesting cases related by Dr. Keiller.|| The essay on puerperal fever by Dr. Flint¶ is an attempt to arrive at useful conclusions, by the application of the numerical method to the analysis of 11 cases of puerperal fever, observed not by the writer himself, but by seven different physicians, who communicated the particulars more or less completely to him.

* Zeitschr. f. d. gesammte Medicin, July, Aug., 1846. † Gaz. Méd., Aug. 2, 1845.
‡ Hoffman, Neue Zeitschr. f. Gebnrtsk., xix, 2tes Heft, p. 194.
§ American Journal of Medical Science, January and April, 1846.
|| Monthly Journal, Feb., 1846. ¶ New York Journal, July,1

Mr. Bell* has reported eight well-observed cases of *pelvic inflammation ending in abscess*. Six of these cases occurred as the immediate sequelæ of parturition; the remaining two happened in women neither pregnant nor recently delivered, but who were exposed to cold while menstruating. There is nothing, however, particularly novel in Mr. Bell's remarks on the affection.

*Lactation*. Two cases of supernumerary nipples are related by Dr. Ashley, and Mr. Garthorn.† In the former instance, they were situated in each axilla, and were connected during suckling with a small tumour the size of an egg, which always disappeared after the woman had weaned her infant. After the patient's third delivery, milk occasionally flowed from these supernumerary nipples. In the other case, both breasts were well formed, but two nipples were situated on the left, about an inch apart, and milk flowed from each of them during suckling.

*Midwifery Statistics, Reports of Hospitals, &c.* The number of deaths in childbirth, as compared with deaths from other causes in this country, appears to be as 1:94.‡ Data do not exist, however, for a statement of its causes, while the great disparity between the returns of different districts, as 1:137, in the South Eastern Division, and 1:65 in the Northern Division, naturally leads to the inference that the returns of death under these circumstances are at present very imperfect.

The note§ contains references to the reports of different hospitals, dispensaries, &c., but all, with the exception of the reports of Dr. Reid and Mr. Earle, and of Professors Götz and Jungmann, refer only to a few hundred cases. Dr. Reid's report contains the result of 1771 cases, and the other three of about 4000 cases each. Their contents, however, are not of such a nature as to allow of abstract within the space of this Report.

### II. On the Progress of Knowledge with Reference to the Diseases of Women.

Comparatively little has been done during the period embraced by this Report in this department of medicine. No new work treating of it has been published, but two more parts of Meissner's elaborate work have appeared. Dr. Rigby's reports on the diseases of women, have been continued in the 'Medical Times,' and many clinical lectures by Dr. Lever have appeared in the 'Medical Gazette;' and some very interesting cases are detailed by Dr. Mickschick‖ in his account of that division of the hospital at Vienna appropriated to the diseases of women.

### DISORDERS OF MENSTRUATION.

*Amenorrhea*. Cases of amenorrhea, the result of an imperforate condition of the hymen, and cured by dividing it, are related by Drs. Barclay and Metcalf.¶ Dr. Krocker, jun.** operated on a girl in whom the menses had never appeared, in consequence of congenital imperforation of the vagina. Peritonitis followed, and proved fatal in 60 hours. It was found on a post-mortem examination that the cervix uteri had become greatly distended, forming a pouch 4½ inches long, by 3¾ broad; but that though no separation existed between the neck of the uterus and its body, that part of the organ was very little, if at all, enlarged, and measured only 2 inches 3 lines in length.

* Med. Gazette, Dec. 12, 1845, Jan. 9, 16, 1846. † Lancet, Aug. 22; ibid., Sept. 12, 1846.

‡ Med. Gazette, Nov. 21, 1845.

§ Mickschick, Oesterr. Med. Jahrb., Oct., 1845; Siebold, Neue Zeitschrift f. Geburtsk., xix, 1stes Heft; Hohl, ibid., xix, 2tes Heft; Hoffman, ibid., 1stes und 2tes Heft; Waddy, Lancet, June 20, 1846; Campbell, Northern Journal, May, 1846; Reid, Med. Gazette, Aug. 15, and Nov. 28, 1845; Earle, Prov. Med. and Surg. Journal, June 10, 1846; Götz, Oesterr. Med. Jahrb., April, 1846; Jungmann, ibid., July, August, September, 1845, and July, August, 1846. ‖ Ibid., Nov., Dec., 1845.

¶ Prov. Med. Journal, Dec. 10, 1845; American Journal of Med. Science, July, 1846.

** Casper's Wochenschr., Oct. 18, 1845.

Two cases of the successful performance of the operation for congenital imperforation of the vagina are recorded by MM. de Bal and Kluyskens.* In each instance, relief was afforded by the first operation, and one of the patients had subsequently menstruated 7 times, while the other, since whose cure many years have elapsed, had married, but never had any family. The writers state that this operation has been performed with success by M. Amussat, as well as by M. Villiaume and M. Manoury, though Boyer and Dehaen both failed, having wounded the bladder instead of opening a passage to the uterus.

*Dysmenorrhea.* The valerianate of zinc, a remedy recently introduced, and reputed to be of great service in some hysterical and neuralgic affections, has been much extolled by Dr. Aldridge† as a cure for certain cases of dysmenorrhea. The dose of the preparation is gr. ¾ to gr. j two or three times a day, in the form either of pill or of solution.

*Menorrhagia.* Mr. Lane‡, who introduced the oxide of silver into medical use, records some cases illustrative of its power in checking hemorrhage from the uterus, whether menorrhagic, or resulting from malignant disease of the organ. The remedy is employed in doses of gr.ss. or gr. j. every 4 or 6 hours, and hemorrhages of a passive nature appear to be those most likely to benefit by it. A similar class of cases appear, from the experience both of Dr. Sée,§ and Dr. Ebers||, to benefit by the use of the ergotine, in gr.ij. doses every 4 hours. The existence of febrile disturbance contraindicates its employment, the stomach under such circumstances often rejecting it. The ergotine is said to be preferable to the ergot of rye, of which it is the essential principle, not merely from its being a much less bulky medicine, but because its long-continued use is found not to be followed by those unpleasant nervous symptoms which have sometimes resulted from perseverance in the administration of the ergot.

### DISEASES OF THE UTERUS.

***Means of investigating them.*** Mr. Fergusson, of King's College,¶ has constituted a speculum which has all the advantages of the ordinary glass speculum, together with others peculiar to itself, while it is quite free from the danger of breaking, which has been urged as an objection to that instrument. It consists of a glass speculum, coated on the outside with silver leaf and varnish, then with cotton cloth, to prevent its breaking, and then with caoutchouc, which gives a smooth external surface. This instrument, which is by no means expensive, presents a very brilliant reflecting surface, not liable to tarnish.

*Displacements of the Uterus.* Dr. Edwards** relates an instance of that rare form of displacement of the uterus, *anteversion.* The patient in whom it existed was not pregnant, and the symptoms, which had come on without any evident cause, had gradually increased in severity. There was no considerable difficulty in defecation, but both pain and difficulty in passing water, attended with very frequent desire to void it. The os uteri could not be brought into view by the speculum, but a large tumour was felt towards the pubis, and it was only by pushing up this body that the os uteri could be brought within reach of the finger; the neck of the womb was large, and directed backwards.

After some preliminary local depletion, and emptying the rectum by means of an enema, the position of the uterus was easily rectified by pushing up the fundus uteri with the index-finger of one hand, and at the same time drawing down the cervix with the fore and middle fingers of the other.

Mr. Gregson†† has added another to the list of successful removals of the

* Gaz. Méd., March 28, 1846.
† Dublin Hosp. Gaz., Aug. 15, Sept. 1, 1845.
‡ Med. Gazette, May 1, 1846.
§ Gaz. Méd., Aug. 8, 1846.
|| Med. Gazette, Jan, 9, 1846.
¶ Ibid., Dec. 19, 1845.
** Lancet, Feb. 7, 1846.
†† Med. Gazette, Feb. 20, 1846.

*inverted uterus* by ligature. The inversion had existed since the patient's delivery two years before, and for the last 18 months she had suffered from constant and profuse hemorrhage. The ligature, which included the whole body and cervix of the uterus, came away on the 9th day, and 3 months after the operation, the patient continued perfectly well.

*Inflammation and ulceration of the os and cervix uteri.* Dr. H. Bennett* has published a series of cases, some of them of considerable interest, illustrative of the views on ulceration of the cervix uteri contained in his recent work on that subject; and some papers on similar topics have been published by MM. Boys de Loury and Costilhes†. M. Pichard‡ attacks what he regards as the too general employment of local cauterization for the cure of various diseases of the os and cervix uteri. The train of argument which he pursues, is to the effect that the cervix uteri being composed of various dissimilar tissues, while the effect of caustics is not confined to the one supposed to be diseased, but extends to all, changes in all result, and any tendency that may exist to cancerous degeneration is thus called into activity. [It cannot be denied that cauterization of the os uteri is sometimes improperly resorted to, and that in other cases a milder treatment would have sufficed for the cure of the patients' ailment. M. Pichard, however, though he speaks of a "host of observations," does not adduce them in confirmation of his statements that cancerous degeneration is often induced by this treatment. In the only case which he relates, where the death of the patient succeeded to cauterization of the cervix, he did not see the patient during the last month of her life, no examination of the body was made after death, and she is reported to have died of carcinoma, only on the evidence of her daughter as reported to M. Pichard by a third party. Hence, although treating of a subject that well deserves careful investigation, the book is in the highest degree unsatisfactory. The remarks on amputation of the cervix contain nothing new.]

Dr. Roberts,§ in two very elaborate essays, treats of *leucorrhea*, and of the importance of employing the speculum in cases where it is present, since it is not a mere increase of the vaginal discharge from debility, but is almost always symptomatic of uterine disease, especially of inflammation of the os and cervix, or more rarely of the lining membrane of the womb. The facts on which his conclusions rest, contain nothing new, but afford a confirmation of the statements of most other writers who have made frequent use of the speculum. M. Gibert‖ strongly recommends an alcoholic tincture of tannin, mixed with seven parts of water as an astringent injection in leucorrhea, and in cases of ulceration of the os uteri. Dr. Mitchell¶ speaks of the employment of the actual cautery to the spine as a means of relieving that extreme pain in the back, which attends some of those cases of leucorrhea where there is very great tenderness of the cervix uteri. It does not appear that he employs the cautery so as to produce a slough, but that he uses it as a mild counter-irritant, similar in its operation to the moxa, though far less severe.

*Polypus uteri.* A paper on this affection has appeared from the pen of Dr. Montgomery,** containing much valuable information. He notices the frequent occurrence of very small uterine polypi, which may not merely escape detection on a vaginal examination, but may even fail to be discovered by the speculum, owing to their being situated between the lips of the os uteri. Even these small polypi, however, are a common cause of ulceration and menorrhagia, the cure of which requires, as a preliminary step, the removal of the polypus. These small polypi derive additional importance from the circum

* In the Lancet for the autumn of 1845, and summer of 1846.

† Gaz. Méd., June, July, August, and September, 1845.

‡ Des Abus de la Cautérisation, et de la Résection du Col dans les Maladies de la Matrice. 8v Paris, 1846.

§ New York Journal, April, July, 1845.

‖ Gaz. Méd., Aug. 9, 1845.

¶ Dublin Medical Press, Oct. 7, 1846.

** Dublin Quarterly Journal, August, 1846.

stance that they are seldom solitary, but for the most part, associated with other kinds of polypi, and especially with fibrous tumour, and that when met with in women at an advanced age, they are often the precursors of some malignant uterine disease. He points out two sources of error, into which the practitioner is likely to fall; the one is the mistaking a tumefied and somewhat elongated condition of the extremity and inner surface of the anterior lip of the os uteri (which sometimes exists in cases of long continued ulceration) for a polypus; the other is the taking the pain and other symptoms attending a polypus for the indications of cancer of the womb; an error in which the practitioner may be confirmed, if he make a hasty examination at a time when a large polypus is in the act of passing through the os uteri. Dr. Montgomery prefers the ligature of polypi to their excision, since, though slower in its action, it usually has the immediate effect of restraining the morbid discharges and alleviating the symptoms, while the hemorrhage, often dangerous after the excision of a large polypus, is sometimes troublesome even when the growth removed is small and its pedicle slender. He adds one important caution, with reference to the management of cases of long standing polypus, attended with copious discharges, which has not been given by previous writers. It is to the effect that after their removal, a condition of the system requiring precaution against determination of blood to the head is likely to follow the suppression of the hemorrhage, to which the patient had become as it were habituated.

Dr. J. H. Bennett* repeats a caution [already given by Lisfranc, 'Clin. Chirurg.' t. iii, p. 210] with reference to the tendency of the wound left after either the ligature or excision of a polypus to degenerate into a troublesome ulceration, and insists on the importance of examining in such cases with the speculum after a patient has recovered from the operation, in order to ascertain whether or no any such ulceration has been left behind. Dr. Montgomery† notices the same fact, but is disposed to attribute the ulceration to the polypus itself, rather than to the means used for its removal. A clinical lecture by M. Lisfranc,‡ on occasion of the death from peritonitis of a patient affected with polypus uteri, in whom the growth had with much difficulty been forced by the uterine efforts through the mouth of the womb, contains some interesting observations. M. Lisfranc remarks that in any case where the symptoms of uterine engorgement, after having been apparently cured, return without evident cause, again disappear under treatment, and once more causelessly return, there is reason for fearing the presence of a polypus. He mentions having seen two or three instances in which the violent expulsive action of the uterus, that is usually regarded as indicative of the presence in its cavity of some body of which it is endeavouring to get rid, occurred although it was quite empty. Lastly, he debates on the conduct to be pursued while the womb is endeavouring to get rid of its contents, which he decides should be merely palliative and expectant; while in those exceptional cases, only three of which have come under his notice where peritonitis followed the expulsion of the polypus from the uterine cavity, all surgical interference must be postponed until the peritonitis is cured. His chief reason for preferring the excision of polypi to their removal by ligature is that the pedicle often being in part composed of uterine substance, there is greater danger of inflammation of the womb following the use of the ligature than of the knife. That the danger of inflammation, however, is not always avoided when the knife is used is shown in the history of a woman aged 42, from whom M. Corni§ removed a large polypus by incision. A severe attack of metro-peritonitis came on on the fifth day, from which the patient recovered only after the employment of very active antiphlogistic measures.

* Lancet, July 19, 1845. † Loc. cit., p. 33.
‡ Gaz. des Hôpitaux, Sept. 15, 1846. § Gazetta Medica di Milano, March 21, 1846.

Dr. Herrich* has endeavoured to improve the operation of excision by inventing a new knife for the purpose, he having been deterred from the use of the ligature as a means of extirpating uterine polypi by a case where the growth being of remarkably dense texture the ligature failed to divide its pedicle, but uterine inflammation was induced, which terminated fatally on the ninth day. He proposes to employ a knife, the stem of which is curved in correspondence with the size of the polypus, while the blade, of a semilunar shape, is placed at right angles to this stem. The blade does not taper towards its extremity, and its only cutting edge, which corresponds to its concavity, is concealed by the same kind of arrangement as that of an ordinary *bistoire caché*, till the knife has been brought to that part of the pedicle which it is intended to divide. The sheath being then withdrawn, and one finger being placed on the extremity of the blade to retain it in the right direction, a slight sawing movement suffices to divide the pedicle. This instrument has been used by Dr. Herrich in two cases, in both of which the polypus had passed through the os uteri, and the scissors might probably have been employed with success. He considers it, however, to be applicable even to cases where the finger cannot be introduced to guide the incision, and the rather, since the cut will always be made at right angles to the body of the polypus and not obliquely, as must be the case when the scissors are employed.

*Malignant diseases of the uterus.* In Dr. Montgomery's paper already referred to, he mentions that a patient from whom he removed a cauliflower excrescence of the uterus by ligature, and whose case he reported in the 'Dublin Journal' for Jan. 1845, still continues well after the lapse of nearly three years.† He likewise relates a second case, where he removed a similar growth by the same means from a patient aged 60. In five days, the growth was detached; the discharges ceased, and the patient's health became perfectly re-established. She continued quite well for four months after the operation; the discharges then began to return, and at the end of another three months the growth had regained its former size, but the patient would not consent to a repetition of the operation. A case is related by Dr. Bodenstab,‡ in which he extirpated the uterus by Langenbeck's operation, by which the peritoneum is not opened. The loss of blood was inconsiderable, but the patient fainted towards the close of the operation, which lasted nearly half an hour, and though she rallied for a short time, syncope came on again, and ended in death, about three quarters of an hour after its completion.

### DISEASES OF THE UTERINE APPENDAGES.

*Diseases of the ovaria.* Dr. Bennett, of Edinburgh,‖ has made some observations on the anatomy and pathology of encysted tumours of the ovary. He notices the doubts that have existed with reference to the source of the fluid found in the abdominal cavity in many cases of this disease, in which it is evidently neither a simple passive effusion, nor the result of inflammatory action. He conceives that in many instances this fluid has originally formed within the ovarian cyst, but escaped from its cavity through some of those ulcerated openings in its walls, that result from the distension of the sac by the accumulation of fluid. Sometimes the adhesions of the cyst to the abdominal walls prevent this occurrence, in which case the only change that takes place is the progressive breaking down of the septa between the different cysts, by which process the dropsical ovary comes in course of time to be composed of one or two large sacs, instead of a number of small cysts either altogether separate from each other, or communicating but very par-

* Ueber Gebärmutter-Polypen, und deren Ausrottung. 8vo, Regensburg, 1846.

† This case is referred to in last year's Report, where, by a typographical error, it is stated that two months had then elapsed since the performance of the operation, instead of 21 months.

‡ Neue Zeitschr. f. Geburtsk., xviii, 2tes Heft. ‖ Edinburgh Med. Surg. Journal, April, 1846.

tially. It is not until the morbid growth has reached this stage that any considerable impairment of the general health begins to take place, an occurrence which he believes to be often symptomatic of the commencement of inflammation and suppuration in the interior of the cyst. He assigns as a reason for postponing tapping as long as possible, the importance of giving time for the occurrence of those changes in the cyst by which it is reduced from a compound to a simple one, since there may then be some ground for hoping that the employment of pressure after tapping may give rise to adhesive inflammation of its walls. He attaches much importance to the uterine sound as a means of discovering the real seat of abdominal tumours, and points out the importance of microscopic examination of the fluid removed from a dropsical ovary as furnishing a clue to the nature of the disease by which it is affected.

A case of permanent cure following the accidental rupture of an ovarian cyst as large as the first is related by Dr. White.* Severe peritonitis followed the accident, and the patient's convalescence was very tedious, but on her recovery the tumour was found to have completely disappeared.

Mr. J. B. Brown and Mr. Hunt,† each relate a case of ovarian dropsy treated by compression, combined with the administration of mercurials and diuretics. The patients are stated to have eventually recovered, though the treatment adopted appears to have placed the lives of both in imminent hazard. In both the mercurial produced most distressing ptyalism, and the diuretics excited painful and almost incessant vomiting. The extremely tight bandaging after tapping induced in Mr. Brown's patient suppuration of the cyst, from which on a repetition of the tapping ten pints of pus escaped. The life of the patient was for many weeks in great danger, but when she got well, the size of the abdomen was found to be much diminished, and the cyst was felt collapsed, hard, and painless. In Mr. Hunt's case the medicines disagreed, the tight bandaging after tapping caused symptoms which could not be relieved except by the removal of the bandage; a step, however, which was not taken until after symptoms of inflammation and fever with delirium had come on. Tapping was performed on Nov. 12; from Nov. 14 to Dec. 3 the patient was very dangerously ill; but on Dec. 9, when she was pronounced convalescent, we are informed that the tumour had entirely disappeared. The results of vaginal examination are not stated, an omission the more to be regretted, since the presence of this tumour in the recto-vaginal pouch two years previously had led Dr. Blundell to sanction the induction of premature labour.

Dr. B. Alison, of Indiana,‡ relates the history of a patient, who having suffered long from ovarian dropsy, and been frequently tapped, was losing her health very rapidly, when he injected a solution of iodine into the sac from which the discharge had for some time been kept constantly flowing by means of a kind of tent that was removed at pleasure. Neither the quantity nor the strength of the solution is stated, but the symptoms which followed its injection are said to have been truly alarming. On their subsidence, however, the patient's health improved greatly, though a little pus still followed the daily withdrawal of the tent. [The introduction of the tent to allow of the daily withdrawal of the fluid resembles the proceeding of Ollenroth mentioned in the Report for 1842-3. The injection of stimulating fluids, suggested by the late Dr. Hamilton, of Edinburgh, though abandoned by him from its bad results, has since been tried by many English and continental surgeons, though generally with unfavorable consequences.]

Mr. Southam and Mr. Dickins§ have extirpated a dropsical ovary with success; the operation not having been succeeded by bad symptoms in either case. Mr. Southam's patient walked a distance of three miles on the nine-

* American Journal, April, 1846, p. 547.
† Lancet, Jan. 10; ibid., Jan. 24, 1846.
‡ Medical Examiner, June, 1846.
§ Prov. Med. Journal, Sept. 10; ibid., Oct. 1, 1845.

teenth day, and Mr. Dickins's patient was up and walking about at the end of three weeks. In both instances the remaining ovary is said to have been quite healthy. Mr. Southam likewise states that the patient on whom he operated in 1843 continues quite well.

Dr. Hayny, Mr. Solly, and Dr. Handisyde,* operated without success. In one of Dr. Hayny's patients it was found, after the abdomen was opened, that the tumour had contracted such extensive adhesions as to render its removal impracticable. In the other the operation was completed, but it was found necessary to remove with the tumour a portion of omentum that adhered to it. The first patient died, apparently exhausted, on the fourth day, the other had several attacks of peritonitis, but lingered on for six weeks, when she died. [In neither instance was any attention paid to the regulation of the temperature at the time of the operation, or to those other points in the general management to which so large share in producing the favorable issue of Dr. Clay's, Mr. Walne's, and other English cases seems attributable.] Mr. Solly's patient died of internal hemorrhage eleven hours after the operation, and Dr. Handisyde's, whose progress was at no time satisfactory, died seventy days after the removal of the ovary from strangulation of the small intestine by a band of lymph encircling it, and which appeared to have been thrown out during the course of three inflammatory attacks after the operation, of which the pelvic viscera showed ample evidence.

### DISEASES OF THE VAGINA AND EXTERNAL ORGANS.

*Vesico-vaginal fistula.* Dr. Zechmeister† relates a case, in which the very frequently repeated introduction of the catheter appears to have been followed by the cure of a fistula of a year's standing. The instrument was at first introduced every hour, afterwards every two or three hours, thus gradually increasing the intervals between the times of employing it. M. Tripet‡ gives a minute detail of M. Bérard's operation for the cure of vesico-vaginal fistula (mentioned in the last Report), by inducing occlusion of the vagina. From the history which he gives of the patient's fatal illness, her death would seem to be scarcelydue to the effects of the operation. A new operation has been suggested for the cure of this accident by M. Jobert§ Its peculiarity consists in detaching a small portion of the vagina by a transverse incision from the cervix uteri before inserting the sutures into the edges of the fistulous opening. The result of this is that the edges are very readily brought into contact, and that all stress upon the sutures is prevented It is said to have succeeded in the three cases in which it has hitherto been tried.

Dr. Oldham‖ has published a fuller account of that *follicular disease of the vulva,* concerning which he contributed a brief notice to Dr. Ashwell's work on diseases of women. [The situation to which he states the disease to be generally limited, namely, two symmetrical strips of mucous membrane at the posterior half of the entrance of the vagina, within the nymphæ, together with the symptoms of itching, and pain on walking, with a thick discharge, seem to point to Duverney's glands as the probable seat of the disease, though Dr. Oldham does not appear to have investigated their condition. The late Dr Fricke, of Hamburg, was the first to call attention to this affection at the scientific congress at Hamburg, in 1838.¶

### III. On the Progress of Knowledge with reference to the Diseases of Children.

A new edition has appeared of Dr. Underwood's well-known work on the diseases of children, edited by Dr. H. Davies, many of whose notes contain

* Oesterr. Med. Jahrb., August, September, 1845; Med. Gazette, July 10, 1846; Edinburgh Med. Surg. Journal, April, 1846.

† Oesterr. Med. Wochenschrift, August 16, 1845. ‡ Arch. Gén. de Méd., September, 1845.

§ Gaz des Hôpitaux, Jan. 24, 1846. ‖ Med. Gazette, May 15, 1846.

¶ A very brief notice of his statements is contained in Rust's Magazin, Bd. xxxiii, p. 143.

very useful practical information. Dr. Coley* has likewise published a work on the same subject, which, while it contains some interesting observations derived from his own experience, is for the most part a compilation from the writings of others. Dr. Friedberg's† treatise on the diagnosis of children's diseases, is entirely a compilation, generally well executed. M. Legendre's‡ volume of essays is a work of much value, consisting indeed chiefly of papers already published, but containing some new contributions, all of which are evidently the fruit of careful and patient observation. The essays which have formerly appeared, are those on hydrocephalus, cerebral hemorrhage, fœtal condition of the lung, and the simultaneous development of variola and vaccinia. The other articles will be noticed in the course of the Report.

### 1. DISEASES OF THE FŒTUS.

Dr. Beatty§ has described a fœtus expelled at the fourth month of pregnancy, the left arm of which was nearly severed by the umbilical cord twisted around it, as has been noticed in other instances of *spontaneous amputation of the limbs in utero.*

Dr. Lasserre|| has related the history of two still-born children, in one of whom *meningeal apoplexy* existed; in the other, which was expelled at five and a half months, there was copious *hemorrhage into the ventricles.* This last fœtus is said to have been dead nearly a fortnight.

Dr. Pastorello¶ saw a child born alive, and living for 2½ hours, whose *hands and feet* were entirely *destitute of epidermis;* the true skin of those parts looking like that of a dead and already putrifying child. The mother is said to have been healthy, and to have previously given birth to healthy twins.

Dr. Götz** describes a case of congenital disease of the skin, which appears to have been the *ichthyosis intra-uterina,* of which instances were mentioned in the last two Reports. The child was a female, born at the full time, of a healthy mother, aged 25 years, in whose pregnancy nothing remarkable had occurred, except that she was much frightened in the 8th week. The fissured condition of the skin, and the eversion of the eyelids were at first very remarkable, and the fissures for some time reappeared after each desquamation. After each, however, the state of the surface improved, and at the time of the child's death, completely atrophied, when 5 weeks old, the skin was merely covered with white scales.

Dr. Schneider†† mentions having twice met with *congenital bronchocele* in children otherwise healthy. In one instance the tumour was as large as a goose's egg, in the other its size was somewhat smaller. Inunction with a weak iodine ointment caused the swellings to disappear in less than 3 weeks. He adds references to similar cases, mentioned by other writers, and extracts from the 'Transactions of the Academy of Sciences at Bologna,' the history of a still-born fœtus, in which an enormous tumour occupied the whole neck, extending downwards towards the sternum, and likewise reaching upwards, especially on the left side, so as greatly to disfigure the face. The tumour was found by M. Mondini, who observed the case to be formed by the enlarged thyroid gland, part of which presented a cellular structure, like that of ordinary bronchocele, while the lower part of the growth more nearly resembled fungus hæmatodes in its characters.

Dr. Hermann‡‡ describes *appearances* which he found *in the lungs* of a fœtus

* A Practical Treatise on the Diseases of Children. 8vo, London, 1846.
† Diagnostik der Kinderkrankheiten. 8vo, Berlin, 1845.
‡ Recherches Anatomo-Pathologiques et Cliniques, sur quelques Maladies de l'Enfance. 8vo, Paris, 1846.
§ Dublin Med. Press, Dec. 24, 1845.
|| Monthly Journal, April, 1846.
¶ Annali Universali, July, 1845.
** Oesterr. Med. Jahrb., June, 1845.
†† Casper's Wochenschr., July 18, 1846.
‡‡ Oesterr. Med. Wochenschr., Feb. 21, 1846.

still-born at the 7th month, resembling what have been described by some writers as *the results of inflammation*, an opinion in which Dr. Hermann coincides. The substance of both lungs was solid and of a reddish brown colour. On their surface, as well as in their substance were numerous small patches, varying from the size of a lentil to that of a bean, of an irregularly round form, and a dirty grayish tint, exceedingly firm and dense, presenting an indistinctly granular structure when divided, and infiltrated with a gray, adhesive matter. [Similar appearances are enumerated at p. 166 of Graetzer's work on 'Diseases of the Fœtus,' who is disposed to regard them as the consequences of inflammation.]

2. GENERAL OBSERVATIONS ON THE DISEASES AND MANAGEMENT OF INFANCY AND CHILDHOOD.

*Diet in infancy.* Dr. Klencke* calls attention to the important deterioration which the milk of stall-fed cows undergoes, and is inclined to attribute the production of scrofula in children in many instances, to its direct transmission through the the medium of that fluid. Although the direct production of scrofula by the contagious properties of the milk is assumed rather than proved in this pamphlet, still the fact is very important that stall-fed cows often become tuberculous, and that their milk loses much or even the whole of its sugar, that the butter and casein diminish, while albumen is found, sometimes in as high a proportion as 15 per cent., and elain in the proportion of 1·4 per cent., and that in some cases lactic acid is likewise present.

In a well-written paper on the subject of diet in children, Dr. Marotte† draws attention to the error often committed in placing infants on a spare diet, who have been observed not to thrive at the breast, but to suffer from diarrhea and to lose flesh. The real means of cure would consist in obtaining a wet-nurse for the child, and thus providing it with a more instead of a less nutritious food. Many instances of gastro-intestinal disorder in childhood depend, in his opinion, on the want of a more highly animalized diet. It is therefore, as a general rule, undesirable to dilute the milk of the herbivora, already poor in animal constituents; while in those cases where it is necessary to supply deficiency in the nutritive qualities of the nurse's milk, chicken or other broth, either alone or mixed with milk, should be used for that purpose.

*Infantile therapeutics.* A manual on this subject has been published by MM. Berton and Schulz.‡ It is decidedly inferior to Dr. Ure's little work on the same subject, which appeared in this country some years ago.

In a paper on the use of opium in childhood, Dr. Sobotka§ takes what seems to the writer of this Report, a very exaggerated view of the dangers of its administration, and expresses opinions which, if generally received, would banish this drug from practice in the cases of children. In many of the cases that he relates as illustrative of the mischievous results produced by opium, diarrhea had existed for some time, and it may be questioned whether the head symptoms were not the consequences of that, rather than of the administration of opium. A very interesting case is related by Dr. M. Barry,‖ of an infant aged 9 months, which had been poisoned by 30 drops of laudanum, and was not seen till seven hours afterwards, when in a state of profound coma. From this state it was roused by the employment of electro-magnetism. At first, when the current ceased for a moment, the child sank into a profound sleep, and there was no marked amendment until the means had been continued for three hours; and four hours and three quarters had passed before

* Ueber die Anstuckung und Verbreitung der Scrofelkrankheit bei Menschen durch den Genuss der Kuhmilch. Leipsig, 1846; and an abstract of it in J. f. Kinderkr., June, 1846.

† Journal de Médecine, August, 1845; and J. f. Kinderkr., March, 1846.

‡ Formulaire Thérapeutique, et Matière Médicale, concernant les Maladies de l'Enfance. 12mo, Paris, 1846.

§ J. f. Kinderkr., Dec., 1845.

‖ Northern Journal, June, 1846.

it was thought prudent to discontinue their use. The child, however, then recovered without any further head symptoms.

### 3. DISEASES OF EARLY INFANCY.

*Asphyxia Neonatorum.* M. Depaul* has written a very elaborate paper on the subject of artificial respiration, as a means of resuscitating still-born children. He instituted a series of experiments on the dead subject, with the view of determining the amount of danger of injuring the lungs by the insufflation of air. He satisfied himself that this danger is almost an imaginary one, since, even after the lungs were removed from the body, it required several most forcible insufflations, far stronger than would ever be made in the case of a still-born child, to produce rupture of the pulmonary vesicles. On the other hand, he was struck with the great force needed thoroughly to inflate the lungs, while their resiliency was sufficient to expel the greater part of the air. He found, moreover, in many cases where children had died suddenly after breathing for several hours or days, no other morbid appearance than an unexpanded condition of a large portion of the lungs. With reference to the mode of practising artificial respiration, he condemns the mere blowing into the mouth as inadequate, and recommends the use of a tracheal tube. He is of opinion that there is more danger of failing from imperfect insufflation, than of doing harm by its too forcible performance. It is of importance, likewise, that it should not be suspended on the first sign of breathing, but continued until the child cries loudly, and respires well.

Dr. Götz† relates a case of the *spontaneous fracture of the left parietal bone* during a natural but tedious labour, in which the head was five hours in the pelvic cavity, although the pelvis was well formed. There were three fissures in the bone; one running into the sagittal suture, one to the anterior inferior angle, and the other to the middle of the anterior edge of the bone. The child was still-born; much blood was effused under the scalp, but none into the skull.

Mr. Close‡ quotes from a manuscript of Dr. W. Hunter's, which he says was published fifteen years ago, an account of *cephalæmatoma,* so complete as to leave nothing to be added to it. Dr. Hunter notices the deceptive sensation of the bone being perforated. He cautions, likewise, against the needless cruelty of opening the tumour, which he says he leaves alone, and it disappears of itself.

*Trismus.* Dr. Sims§ calls attention to the intense vascularity of the vessels of the spinal cord, and the effusion of blood around it, often met with in cases of trismus, and observed by him in the instance of which he has recorded the dissection. He endeavours to account for the spinal apoplexy, by assuming that the new-born child lying on its back, with a hard support beneath its yielding skull, the edge of the occipital bone is driven up under the parietal bones, and the cerebral circulation is thus interrupted. [It is scarcely necessary to remark, that any such mechanical theory of the disease is contradicted by the remarkable influence of climate in the production of the disease, and by such facts as its extreme frequency at one time in the Dublin Lying-in Hospital, and its almost complete disappearance after an efficient system of ventilation had been adopted.]

*Spina bifida.* Dr. L. de Thinnecour|| relates the history of the successful treatment of a case of this affection in a child aged two months, in which a tumour of the size of an infant's head grew from the junction of the lumbar and sacral vertebræ. Instead of employing a common circular ligature, he used an instrument that acted on the principle of Dupuytren's *enterotome,* then punctured the sac, and afterwards laid it open through its whole extent. In ten days the communication with the vertebral column was closed, and he

* Journal de Chirurgie, May, June, 1845; and J. f. Kinderkr., März, 1846.

† Oesterr. Med. Jahrb., April, 1846.

‡ Med. Times, Sept. 20, 1846.

§ American Journal of Med. Science, April, 1846.

|| J. f. Kinderkr., May, June, 1846.

now removed the instrument, and placed a common circular ligature round the pedicle. The wound granulated and healed kindly, and at nine months old the child was perfectly well, and had had no return of the disease. The advantages of this mode of operating he conceives to be, that the membranes of the spinal cord are secured from the access of air after the puncture, that the two rods on each side of the spine form a substitute for the arches of the vertebræ, and favour the union of the serous surfaces, while they also help to diminish the space between the arches themselves. Dr. Beaunier† employed the common ligature in a child ten days old, with a spina bifida proceeding from the third cervical vertebra. Having tied it, he punctured it twice. Having tightened the ligature after the second puncture, the cyst began to become gangrenous, whereupon he cut it off. The wound healed, and four months afterwards the child was well.

Dr. Williamson‡ has described a very interesting case of *imperforate anus,* in which the rectum terminated in the urethra, the child, nevertheless, living for 8 months, and for 5 months of this time passing its fæces with moderate ease by the urethra. About that time, however, the child began to take other food besides the mother's milk, its fæces became more solid, and escaped with greater difficulty. Attacks of constipation of increasing severity now began to recur frequently, and in one of these the child died. The communication was found to exist at the membranous portion of the urethra, by means of a canal a quarter of an inch in diameter, and half an inch long, while the gut terminated in a blind pouch. Dr. Williamson made an unsuccessful attempt during the life of the child to open the rectum, but was not allowed to repeat it. He adds a caution against carrying the trocar too far backwards in operating in cases of this kind, since by so doing, the instrument may pass behind the rectum instead of puncturing it.

Dr. Thore's‡ observations on *peritonitis* in new-born children contain much valuable information. The general characters of the disease appear to be the same as it presents in the fœtus, and the same absence of puriform fluid in the abdominal cavity is noticed here. A dirty serous fluid, with fibrinous flocculi floating in it, is often observed, and layers of pale membrane cover the intestines, and are especially abundant about the spleen and liver. In one third of the cases, 63 in number, pleurisy was found associated with the peritonitis, another point in which it resembles the disease as it occurs in the fœtus. It appears, moreover, that this affection is most frequent during the first fortnight of existence, while after the first month it is very rare; a circumstance which suggests the doubt whether it does not, in some instances, commence even before birth. In several cases, however, this certainly was not so, for M. Thore found that the season of the year had much to do with the prevalence of the disease, and that it is most frequent during the spring and summer season, partly, perhaps, owing to the wards of the hospital being then most crowded. He inclines also to the opinion that those conditions which favour puerperal fever likewise increase the frequency of peritonitis in the infant; and he establishes, conclusively, the existence of a relation between infantile erysipelas and peritonitis, since 17 of 26 cases of erysipelas were combined with peritoneal inflammation, and a similar relation may be noticed between peritonitis and phlebitis of the umbilical vein.

A sudden, tympanitic swelling of the abdomen is often the first symptom of the disease, and is soon associated with vomiting of a greenish matter, which phenomenon, however, is seldom of long continuance. The bowels are generally constipated throughout, the respiration and pulse become accelerated, the heat of the skin is increased, and the child evidently suffers pain in the abdomen. As the disease advances the countenance alters, the skin grows cold, and the pulse feeble, the child dying, in the great majority of cases, in

* J. f. Kinderkr., July, 1846. † Med. Gazette, May 1, 8, 1846.

‡ Arch. Gén. de Méd., Aug. and Sept., 1846.

less than twenty-four hours, and no instance having ever come under his notice where the symptoms ran a chronic course. All the cases that M. Thore saw terminated fatally, his recommendation, therefore, of depletion and calomel rests on theoretical grounds, not on any well-ascertained beneficial results of their employment.

### 4. DISEASES OF SUBSEQUENT CHILDHOOD.

#### DISEASES OF THE BRAIN, NERVOUS SYSTEM, ETC.

An interesting case of the occurrence of *apoplexy*, in a healthy boy, aged 11 years, is recorded by Mr. Worthington.* The right ventricle was full of extravasated blood, and the brain around lacerated to a considerable extent, but no ruptured vessel was distinguishable. When first seen after the attack, the child was extremely faint and exhausted, but paralysis was not present, though the right pupil was extremely dilated, and the left much contracted. General convulsions occurred every ten minutes for the last ten hours of the child's life, and he died in one of these fits, about twelve hours after the seizure.

*Inflammatory affections.* A work has been written by Dr. d'Alnoncourt,† addressed to the public at least as much as to the profession, in which he combats the generally-received opinion of the frequency of inflammatory affections of the brain, especially at the time of dentition, and refers almost all the diseases of childhood to impairment of nutrition and disorder of the digestive organs. The book abounds in exaggerated statements, and declamations against physicians, but contains no new facts, and no record of observations.

Dr. Mayne‡ has given a brief sketch of an epidemic of *cerebro-spinal arachnitis*, which has recently prevailed in the Irish workhouses, and in some of the Dublin hospitals. It resembled, both in its symptoms and in the morbid appearances to which it gave rise, the affection that was epidemic in many parts of France, from the year 1840 to 1842. The arachnoid was found in every case extensively inflamed, and lymph was poured out beneath it; the arachnoid of the spinal cord being in every instance much more severely affected than that of the brain, while the nervous substance was comparatively seldom involved, and never in any considerable degree. In France the young conscripts were most frequently attacked, and in Ireland its most frequent subjects were boys under 12 years of age, while there, as on the continent, females very seldom suffered from it. Its attack was generally very sudden, and its course often extremely rapid, some patients dying in twenty-four hours, while few survived the 4th day. Severe pain in the abdomen, attended with vomiting and purging, and a condition of general collapse, marked the outset of the disease. A stage of reaction followed this in a few hours, the surface becoming hot, the pulse full, and its frequency varying from 120 to 140, while the face assumed a tetanic expression, and the head was retracted and firmly fixed. General convulsions or coma succeeded to this condition, and failure of deglutition, and a slow and laboured pulse soon followed as the immediate precursors of death. Dr. Mayne does not state the exact number of cases that came under his care, but he mentions that he, in common with other physicians, often found treatment of no avail, though he seems to have employed depletion and mercurials with great decision in those cases which he saw early.

*Acute hydrocephalus.* In a paper on the premonitory symptoms of this disease, M. Rilliet§ expresses his dissent from the opinion of those writers who have attributed them either to the occurrence of small effusions of fluid into the ventricles, or to a congested state of the brain, or to a chronic meningitis ushering in the more acute affection. He regards the so-called premoni-

* Prov. Med. Journal, April 22, 1846.

† Die Gehirnaffectionen der Kindern in der Dentitionsperiode, &c., eine Täuschung der Aerzte. 8vo, Leipsic, 1846. ‡ Dublin Quarterly Journal, Aug., 1846. § Gaz. Méd., Jan. 1846.

tory symptoms of the disease as the index of the general tuberculization, rather as the sign of the local deposit of tubercle in the membranes of the brain. The child shows signs of ill health when the lungs and bronchial glands are becoming the seat of the morbid deposit, and it does not always happen that these premonitory signs are followed by the outbreak of the acute disease, for sometimes they pass into confirmed phthisis, while in other instances they altogether disappear under appropriate treatment. He states, moreover, that the general deposit of tubercle is more abundant, and it is found to have reached a more advanced stage directly in proportion to the longer duration of the premonitory symptoms, while, on the other hand, their duration and that of the meningitis are in an inverse ratio to each other.

A series of articles have appeared from the pen of Dr. Bierbaum,* on the diagnostic value of the different symptoms of acute hydrocephalus. They are by no means destitute of merit, though far inferior to the pamphlet on the same subject, published some years ago by Dr. Wolff, of Bonn.

*Chronic hydrocephalus.* Dr. Spengler† has published an analysis of the fluid from a chronic hydrocephalus, from which he concludes that at any rate, in this instance, it was not the product of inflammatory action, but was the result of the morbid accumulation of the cerebro-spinal fluid.

Dr. Edwards‡ mentions the case of a child who recovered from chronic hydrocephalus after a single tapping. This proceeding was adopted when the child was 14 months old, after six months of unsuccessful treatment. Eight ounces of a rose-coloured fluid were drawn off, and the child recovered, and continued well at the end of seven years. Dr. Götz and Mr. Chater§ have punctured the brain unsuccessfully. Both children were 5 months old, and in both the disease was congenital. In the former case three punctures were made within eighteen days; temporary amendment succeeding to each, but the fluid collected again rapidly, and death in convulsions took place five days after the last puncture. Mr. Chater modified the operation by introducing two or three threads into the puncture in order to act as a syphon, and thus keep up a constant drain from the brain. By this plan the head was reduced 5½ inches in circumference in five weeks; the child then suddenly grew comatose and died. The brain was in this case so extensively disorganized, as to have rendered cure quite impossible, but the practice of Mr. Chater deserves another trial. [There are now 63 authenticated cases on record in which puncture of the brain was performed; and in 18, or 28·5 per cent. of these the child recovered.]

DISEASES OF THE ORGANS OF RESPIRATION.

A paper has been published by Dr. G. A. Rees,|| 'On Carnification of the Lungs in Infants,' which might be dismissed without further notice, if it were not that in it he asserts a claim to have been the first who described this condition, and insinuates a charge of plagiarism against MM. Rilliet and Barthez, who, he says, in their work on Chidren's Diseases almost quote his own words. [The title of the paper on which Dr. Rees founds his claim, is 'On deformity of the chest in young children from disease of the lungs;' and it was published in the 'Medical Gazette' for January 12, 1839. It is fortunately unnecessary to analyse this paper in order to vindicate MM. Rilliet and Barthez from this singular accusation, since the article pneumonia in their large work on Children's Diseases, is little else than a reprint of their monograph on that subject which appeared in 1838.¶ In that monograph will be found the particulars of 11 observations of the condition which they designated as carnification of the lungs, as well as the very words between

* J. f. Kinderkr., July, Aug., Sept., 1846.

† Oesterr. Med. Wochenschr., July 12, 1845.

‡ Monthly Journal, June, 1846.

§ Oesterr. Med. Jahrb., June, 1846; Prov. Med. Journal, Oct. 1, 1845.

|| Lancet, July 11, 1846.

¶ The title of this work is, Maladies des Enfans. Affections de Poitrine. Première Partie—Pneumonie. Par MM. Rilliet et Barthez. 8vo, Paris, 1838.

which and his own expressions, published some months afterwards, Dr. Rees professes to discover so remarkable a coincidence.]

DISEASES OF THE ORGANS OF DIGESTION, AND ASSIMILATION, AND THEIR APPENDAGES.

Dr. Panck* has described an epidemic of *cynanche parotidea,* that prevailed in the Alexandrine Orphan House at Moscow in the commencement of the year 1840, and affected 162 out of 300 inmates between the beginning of January and the beginning of April. Both sexes appeared to be equally liable to the disease, but young persons about the age of puberty were most frequently attacked by it, while children under 7 years old had simple febrile seizures, without any swelling of the glands. It was quite evident that some epidemic influence was concerned in its production, and that it did not arise simply from cold; neither did cold appear to be the principal cause of metastasis of the swelling from the parotids to other parts, though it did sometimes seem to cause a relapse in patients who had appeared convalescent.

Dr. Duncan† relates several cases of *ulcerous stomatitis,* connected with diarrhea and entero-colitis, which he observed among the children of the South Dublin Workhouse. He gives a brief but good account of the disease, and dwells at some length on the evidence of its not being dependent on the administration of mercurials, though it would seem, from his remarks, that he regards it as identical with cancrum oris, an opinion to which the writer of this Report cannot by any means subscribe.

A very interesting account has been given by Dr. Daviot of an epidemic of *diphtheritis,* which prevailed in the department of the Saône and Loire from 1841 to 1844.‡

He states that the disease never occurred sporadically in this district, and that no case of it had been observed since 1809; though between 1782 and 1809, it had been four times epidemic.

The pharynx was the part most frequently affected by it, and next to that the skin; then the mucous membrane of the trachea and larynx, and lastly, that of the mouth; but it frequently attacked many parts simultaneously. The affection of the skin often came on with intense redness, followed by ulceration; or the skin appeared excoriated, and these excoriations then became covered with lymph. In other instances, an eruption resembling scarlatina appeared over the whole surface, although not followed by any diphtheritic affection of the skin.

The course of the disease was very rapid. It reached a high degree of severity in from 36 to 48 hours, and its fatal termination took place in from 7 to 10 days; the patients dying asphyxiated. Sometimes, after apparent recovery, bronchiopneumonia supervened, and led to a fatal result.

Dr. Daviot bled both generally and locally at the onset of the disease. In the first stage he employed alum locally, but as the disease advanced, he had recourse to the nitrate of silver, believing it to be more energetic than hydrochloric acid. Rubefacients were frequently applied to the throat with advantage, but blisters were avoided, on account of the tendency to diphtheritic affection of the skin.

*Diarrhea.* The work of M. Legendre§ contains an essay on this subject, founded on the observation of 28 fatal cases. He ascertained that in some instances no alteration whatever of the intestinal mucous membrane could be found, while in the majority of cases enlargement of the intestinal follicles, or their more or less extensive ulceration, constituted all the morbid appearances. These changes of the follicles, too, appear always to precede any alteration of the mucous membrane itself. From these facts he concludes

* Zeitschr. f. d. gesammte Medicin, Sept., 1845. † Dublin Journal., Sept., 1845.

‡ The writer has been unable to obtain this pamphlet, which was published at Autun, in 1845; but an abstract of it is given in the J. f. Kinderkr., June, 1846. § Op. cit., pp. 363-418.

that the diarrhea of early childhood is at first merely an excessive secretion, and not the result of any appreciable morbid change, and that the anatomical alterations of the digestive canal are the consequences of the diarrhea, not its cause, whence it happens that their extent is usually proportionate to the severity and continuance of the flux. The grand object of this essay is to combat the opinions of the disciples of Broussais, and by disproving the inflammatory origin of diarrhea, to explain the frequent failure of antiphlogistic treatment, and to justify a return to the practice of the old physicians, and the use of evacuants and absorbents.

Dr. G. Bird* has made an *analysis of the green evacuations of children.* He finds that they give no indication of containing an excess of bile, as has generally been supposed, but conceives that their colour is due to the presence of altered blood, and that the state in which they are produced is analogous to that which exists in melæna, the portal system generally being in a congested state. In support of his views, he mentions that stools originally of a yellow or orange colour, often turn green under exposure to air, an occurrence very unlike what takes place with matters containing bile, though it is an ascertained fact that blood under the influence of some oxidating agents acquires a green colour.

Two cases of fatal *intussusception of the intestines* are related; the one by Dr. Boyer, the other by Mr. Markwick,† in each of which the characteristic symptoms were present.

Dr. Duclos‡ relates two cases of *fissure of the anus* in infants, treated by M. Trousseau. The symptoms seem to be the same as in the adult, namely, pain and spasmodic contraction of the sphincter, betokened by a cry when the bowels act, and the escape of a drop or two of blood. M. Trousseau treats these cases successfully with enemata of one part of extract of krameria to 100 of water.

M. Morand§ recommends the extract of belladonna as a valuable remedy for *nocturnal incontinence of urine*, in those cases of it which seem to be associated with a state of general debility. For children from 4 to 6 years old, he begins with a pill containing gr. $\frac{1}{4}$ of the extract twice a day, increasing the dose to gr. j in the course of 14 days. He suspends the medicine if symptoms of narcotization come on, but otherwise continues it for 2 or 3 months so as to effect a perfect cure.

### FEVERS.

*Ague.* Dr. Petzold,|| who inhabits a malarious district, describes the peculiarities which this disease presents in early childhood. Its characters are on the whole less marked, so that there is some danger of mistaking the nature of the affection. The shivering fit is less severe, and neither the hot nor the sweating stage is so well marked as in the adult. The intermissions, likewise, are less complete, the child being manifestly out of health between the paroxysms, while there is often a very great tendency in the attack to anticipate, so that the periodicity of its return may easily be lost sight of.

When it occurs in children of only a few months old, the cold stage usually sets in very suddenly, attended with great depression, or sometimes it comes on with convulsions, and manifest cerebral disturbance, so that when the hot stage has succeeded, the case may be taken for one of inflammation of the brain. The tranquil sleep, however, into which the child falls as the hot stage passes off, will serve to guard from this error. It is of importance to recognize the disease early in the infant, as well as in the aged, since the attacks of ague exhaust the strength very rapidly, while quinine, which is of the most marked benefit while the disease retains the intermittent type, seems to lose much of its efficacy so soon as the fever has assumed the continued form.

* Med. Gaz., Sept. 5, 1845. † Casper's Wochenschr., March 10, 1846; Lancet, July 18, 1846.
‡ J. f. Kinderkr. June, 1846. § Ibid., Sept. 1845. || Ibid., Sept. 1845.

*Typhoid Fever.* Dr. Löschner,* physician to the Children's Hospital at Prague, has written a paper on this subject, the materials for which are drawn from the observation of 104 cases that came under his notice among a total of 6500 children, at a time when fever was not epidemic. He ascertained the ages from 5 to 9 to be those during which the disease is most prevalent, while it attacks boys more frequently than girls. The mortality among the cases that he observed was 8 in 104. He notices a greatly enlarged and highly injected state of the mesenteric glands as having been much more constant than ulceration of Peyer's glands; and on this tendency to affection of the glands, he builds the hypothesis that what is called typhoid fever, is a kind of acute scrofula, though he adduces no other fact in support of this theory.

*Measles.* An epidemic of this disease that prevailed in the neighbourhood of Glogau, in the spring of 1843, is described by Dr. Posner.† The disease did not cause any remarkable mortality, and presented nothing unusual in its course.

Dr. Battersby‡ has related some very interesting cases illustrative of the complications and sequelæ of measles, as he observed them during an epidemic in the autumn of 1844. The affection presented much of an asthenic character, and was often associated with diarrhea and dysentery, and with inflammation of the mouth and pharynx. In two or three cases also, where the powers of life were much exhausted, sloughing of the cornea took place. Unlike the affection of the eyes which comes on in phlebitis, it was attended with but very little increase of vascularity; [and it seems questionable whether it was not due rather to the general impairment of nutrition than to any specific influence of the poison of measles.]

*Scarlatina.* The pamphlet of Mr. J. B. Brown§ on this subject has been so generally noticed in the various medical journals, as to render any further mention of it in this Report unnecessary.

Dr. Merbach‖ has described the *dropsy that succeeded to scarlatina,* in an epidemic at Dresden, and which sequela appears to have been extremely fatal, causing the death of nearly 1 in every 3 who suffered from it. The treatment adopted, which consisted chiefly in the administration of stimulant diuretics, with the neglect of depletion and of all decided antiphlogistic means, will probably in some measure account for this mortality. Dr. Merbach confirms the statements of previous observers with reference to the characters of the urine, and the fluctuations in the quantity of albumen it contained without any apparent cause. He notices, moreover, that the diminution in the quantity of urea was always in direct proportion to the abundance of the albumen, but that the increase in the quantity of the former always took place more slowly than the diminution of the latter. The work of M. Legendre¶ contains a valuable essay on the *anasarca and the œdema of the lung* which occasionally succeed to scarlatina. He first notices the frequency with which the eruption of the scarlet fever is overlooked, in consequence of its being but very temporary, and insists on the importance of making very minute inquiries and examining the surface very carefully in all the febrile affections of childhood, in order to ascertain whether the rash is or has been present. He next lays down rules for the hygienic management of children during their convalescence from scarlatina. Lastly, he inquires into the nature and causes of the dropsy, which he regards as the simple result of the action of cold, and not as the consequence of renal disease; the albuminous state of the urine being in his opinion produced by a simple nephritis or even by a congested state of the kidney, and not the token of an incipient stage of Bright's disease. In support of this opinion he appeals to

* J. f. Kinderkr., Dec. 1845. † Ibid., Sept. 1846. ‡ Dublin Journal, Sept. 1845.

§ On Scarlatina and its successful Treatment by the Acidum Aceticum Dilutum of the Pharmacopœia. 8vo, London, 1846. ‖ J. f. Kinderkr., May, 1846. ¶ Op. cit., pp. 305-362.

the connexion between the quantity of albumen and that of blood in the urine, and to the simultaneous diminution in the two as the patient approaches towards convalescence. The number of cases on which M. Legendre's remarks are based was only 14; and he does not seem to have had the opportunity of watching any patients who died after the disease had reached a chronic stage, so as to determine whether any tendency to granular degeneration of the kidney is induced by the previous scarlatinal dropsy. [The correctness of his views, however, is borne out by the observations of cases where the dropsy has existed unconnected with albuminous urine, as in the epidemic at Berlin, in the spring of 1840,* as well as by the results of recent microscopic investigations, such as those of Henle, Eichholtz, and Dr. G. Johnson.]

The concluding part of his essay gives an account of that œdema of the lung which comes on as a sequela of scarlatina, for the most part in cases where general anasarca is present or has previously existed. He describes the sudden manner in which its symptoms often appear, while though the dyspnœa that attends it is extremely urgent, there are no auscultatory signs of the affection of the lungs. The chief point in the paper. however, is the anatomical description of this condition, which is an œdema of the interlobular celluar tissue, compressing the air-cells in greater or less degree, and thus differing from the œdema of Laennec, in which the fluid is supposed by that author to be contained within the pulmonary vesicles.

*Variola.* An elaborate paper on the *anatomy of the smallpox pustule* has been written by Dr. Simon,† of which only brief mention can be made here. He states that the pustule does not always owe its central depression to the presence of a hair-follicle pinning down the epidermis, but that in parts where no hair-follicle exists, the appearance is probably owing to the rapid desiccation of the exudation first poured out, while fresh matter is afterwards effused around it. The white membraniform layer immediately below the surface of the pustule is not in reality a false membrane, but is chiefly made up of the lower desintegrated stratum of epidermis, and the cellular structure of the pustule is produced by this stratum remaining in connexion with the cutis at some points, while at others it is detached from it.

The utility of the application of *mercurial ointment* or plaster, as a means of producing the *abortion of the smallpox pustule,* and thus preventing pitting, and diminishing the danger of the disease, is confirmed by the experience of MM. Goblin, Charcellay, and Briquet,‡ and M. Thielmann and Dr. Panck§ state that they have obtained equally favorable results from the frequent use of a solution of the corrosive sublimate. M. Thielmann employed it of the strength of gr.j to ℥ij. Dr. Panck used it of about half that strength.

M. Tardieu‖ has related a case of the *simultaneous existence of variola and vaccinia* in a man aged 18, who was vaccinated on the day on which the eruption of smallpox had made its appearance. The variola ran its course with its characters modified, and after the desquamation of its pustules an irregular eruption of cowpox appeared. From this case he concludes that we may vaccinate with the hope of doing good, not merely during the preliminary fever of variola, but even after the outbreak of the eruption.

The work of M. Steinbrenner,¶ who, with M. Bousquet and M. Fiard, has shared the prize of the French Academy for the best essay on *vaccination* and *its influence on smallpox,* will be found to contain a great amount of information on the subject, collected with the most laborious industry. In reply to the five questions proposed by the Institute, M. Steinbrenner decides—

* Described by Dr. Philip, in Casper's Wochenschr., Aug. 29, 1840. † Müller's Archiv, 1846, ii.

‡ Revue Méd., and Oesterr. Med. Wochenschr., September 20, 1845; Bull. de l'Acad. Roy. de Méd., April 15, 1846; Gazette des Hôpitaux, September 19, 1846.

§ Gazette des Hôpitaux, April 16, 1846; Oesterr. Med. Wochenschr., September 20, 1845.

‖ Gaz. Med., November, 1845. ¶ Traité sur la Vaccine. 8vo, Paris, 1846.

1st. That the preservative power of vaccination is almost always permanent, and that when it is not so the period of immunity varies greatly according to individual peculiarities. 2d. That the vaccine virus does undergo a positive deterioration by transmission through successive individuals. 3d. It is therefore desirable to obtain fresh lymph frequently, which might be done by taking it annually from the cow, by which we should be much more sure of succeeding than by retrovaccination or any similar means. 4th. There is no necessary connexion between the intensity of the local phenomena of vaccination and its preservative power, but there is such a relation between the preservative power and the amount of constitutional disturbance. 5th. Revaccination is desirable not because it is always necessary, but because we have no means of distinguishing the cases where it is needed from those in which it is superfluous.

A very elaborate collection of statistics, intended to illustrate the same questions as are treated of by Dr. Steinbrenner, has been made by Dr. Lane,* but is not of a kind to admit of abstract.

In an account of an *epidemic of smallpox at Heidelberg*, and of revaccinations which he practised there, Dr. Hoefle† makes an assertion which is opposed to general experience. He asserts that he found the pustules of revaccination bear to those of primary vaccination just the same relation as those of a second attack of variola bear to those of a first attack. He states, moreover, that he observed this modification, although he never employed revaccine lymph, and though he always vaccinated directly from arm to arm.

M. Legendre‡ has related the particulars of some *chronic affections of the skin* which were *cured by* the appearance of the *eruption of smallpox*. The cases which underwent improvement were either papular, vesicular, or pustular, while an eruption of porrigo favosa of the scalp was not in the least benefited by a copious eruption of smallpox.

### DYSCRASIÆ, ETC.

*Gangrene.* Dr. Battersby§ describes a case of gangrene of the skin in a female child, aged 10 months. The disease began with the appearance on the limbs of several vesicles, a good deal like those of varicella, but larger, the cutis beneath some of them being black and gangrenous. The child lived for a fortnight, during which time no attempt took place at separation of the dead parts, and the gangrene extended from the thigh to the vulva, and partly up the abdomen. Dr. Battersby mentions a similar case recorded by Dr. Hutton in 'Dublin Journal,' xvii. p. 485. [A case is mentioned by Rilliet and Barthez, 'Maladies des Enfans,' ii. p. 195; references to others are given by Richter, 'Ueber den Brand der Kinder,' pp. 9-12; and one instance of it came under the notice of the writer of this Report, in which the skin of the face was affected.]

*Scrofula.* Mr. Phillips's Treatise on Scrofula ‖ contains a very large amount of valuable statistical information. The grand objects of the work, however, is to prove the non-identity of phthisis and scrofula. He confesses the apparent identity of the deposit, however tested; but in scrofula inflammatory change in the gland precedes the deposit; while the lung is unaltered around a simple deposit of tubercle. Further, the two diseases are not prevalent in the same districts, nor in the same sex, nor at the same age, and 18 out of 20 phthisical patients show no sign of scrofula; from all which facts taken together Mr. Phillips draws the conclusion that though allied they are not identical diseases.

* American Journal of Med. Sci., July, 1846.

† Gaz. Méd., April 25, 1846.

‡ Op. cit., p. 439-449.

§ Dublin Hospital Gazette, March 15, 1846.

‖ Scrofula,—its Nature, its Causes, its Prevalence, and the Principles of Treatment. 8vo, London, 1846.

